# Cholinergic and Neuroimmune Signaling Interact to Impact Adult Hippocampal Neurogenesis and Alcohol Pathology Across Development

**DOI:** 10.3389/fphar.2022.849997

**Published:** 2022-03-02

**Authors:** Victoria A. Macht, Ryan P. Vetreno, Fulton T. Crews

**Affiliations:** ^1^ Bowles Center for Alcohol Studies, School of Medicine, University of North Carolina at Chapel Hill, Chapel Hill, NC, United States; ^2^ Department of Psychiatry, School of Medicine, University of North Carolina at Chapel Hill, Chapel Hill, NC, United States; ^3^ Department of Pharmacology, School of Medicine, University of North Carolina at Chapel Hill, Chapel Hill, NC, United States

**Keywords:** ethanol, hippocampus, acetylcholine, fetal alcohol, adolescence, choline, cytokines, doublecortin

## Abstract

Alcohol (ethanol) use and misuse is a costly societal issue that can affect an individual across the lifespan. Alcohol use and misuse typically initiates during adolescence and generally continues into adulthood. Not only is alcohol the most widely abused drug by adolescents, but it is also one of the most widely abused drugs in the world. In fact, high rates of maternal drinking make developmental ethanol exposure the most preventable cause of neurological deficits in the Western world. Preclinical studies have determined that one of the most consistent effects of ethanol is its disruption of hippocampal neurogenesis. However, the severity, persistence, and reversibility of ethanol’s effects on hippocampal neurogenesis are dependent on developmental stage of exposure and age at assessment. Complicating the neurodevelopmental effects of ethanol is the concurrent development and maturation of neuromodulatory systems which regulate neurogenesis, particularly the cholinergic system. Cholinergic signaling in the hippocampus directly regulates hippocampal neurogenesis through muscarinic and nicotinic receptor actions and indirectly regulates neurogenesis by providing anti-inflammatory regulatory control over the hippocampal environmental milieu. Therefore, this review aims to evaluate how shifting maturational patterns of the cholinergic system and its regulation of neuroimmune signaling impact ethanol’s effects on adult neurogenesis. For example, perinatal ethanol exposure decreases basal forebrain cholinergic neuron populations, resulting in long-term developmental disruptions to the hippocampus that persist into adulthood. Exaggerated neuroimmune responses and disruptions in adult hippocampal neurogenesis are evident after environmental, developmental, and pharmacological challenges, suggesting that perinatal ethanol exposure induces neurogenic deficits in adulthood that can be unmasked under conditions that strain neural and immune function. Similarly, adolescent ethanol exposure persistently decreases basal forebrain cholinergic neuron populations, increases hippocampal neuroimmune gene expression, and decreases hippocampal neurogenesis in adulthood. The effects of neither perinatal nor adolescent ethanol are mitigated by abstinence whereas adult ethanol exposure-induced reductions in hippocampal neurogenesis are restored following abstinence, suggesting that ethanol-induced alterations in neurogenesis and reversibility are dependent upon the developmental period. Thus, the focus of this review is an examination of how ethanol exposure across critical developmental periods disrupts maturation of cholinergic and neuroinflammatory systems to differentially affect hippocampal neurogenesis in adulthood.

## Introduction

The birth, maturation, and functional integration of new neurons, termed neurogenesis, is a critical developmental process originally thought to be isolated to fetal and early neonatal development wherein bursts of new neurons aggregate to form the various regions of the central nervous system (Altman and Das, 1965). In humans, neurogenesis associated with the developmental formation of the brain occurs across gestation, with cortical structures forming prior to the hippocampus and the hippocampal dentate gyrus not demonstrating mature cytoarchitecture until 34 weeks, making it one of the last structures to mature in humans (Arnold and Trojanowski, 1996) and rodents (for review *see* (Snyder, 2019)). This rapid maturation of the human nervous system during the human third trimester of pregnancy has been colloquially termed the “brain growth spurt” and corresponds to the first 10 days of postnatal life in rodents, where rodent hippocampal development similarly peaks (West et al., 1986). The developmental view that neurogenesis terminates near birth in humans and in the early neonatal period in rodents theoretically left mammals with a finite number of neurons for the duration of their lifespan and led to the erroneous conclusion that any subsequent loss of neurons through drug use, stress, traumatic brain injury, or insult was permanent. However, this dogma has been challenged over the last several decades with emerging evidence indicating that select mammalian brain regions continue to generate and functionally integrate new neurons to varying degrees throughout the lifespan (for review *see* (Gross, 2000; Ming and Song, 2005)). One region of continuous neurogenesis is the subventricular zone of the lateral ventricles; the other, which is the focus of the current review, is the subgranular zone of the hippocampal dentate gyrus (Altman and Das, 1965). It is important to note that while this review will focus on findings surrounding markers of adult neurogenesis from rodent studies, the prevalence of adult neurogenesis in humans remains an ongoing scientific debate. For example, while doublecortin is a conventional marker of neurogenesis in rodents, recent single-nucleus RNA-seq data question its validity as a comparable marker of adult neurogenesis in humans (Franjic et al., 2022). These findings highlight the center of this debate, which predominantly consists of technical issues in identification and labeling of adult newborn neurons in humans. See Kempermann et al. (2018) for more insight regarding this critical discussion.

In contrast, functional integration of adult hippocampal newborn neurons in rodents has been critically linked to brain health, plasticity, and cognitive function, broadly, but also more specifically to discrete roles in spatial processing and pattern separation (Clelland et al., 2009) as well as cognitive flexibility (Anacker and Hen, 2017) and reversal learning (Garthe and Kempermann, 2013). The unique role of adult hippocampal neurogenesis in cellular and behavioral plasticity is supported by evidence that loss of neurogenesis, which occurs with age as well as with exposure to stress, drugs, or disease, is tightly coupled to loss of cognitive function in these domains (Burghardt et al., 2012; Winner and Winkler, 2015; Anacker and Hen, 2017). Similarly, restoration of hippocampal neurogenesis through lifestyle or therapeutic interventions can recover cognitive functioning (Abdipranoto et al., 2008). As such, restoration of neurogenesis has emerged as a central factor to be considered in various cognitive therapeutic interventions (Abdipranoto et al., 2008).

Disentangling the mediators of hippocampal neurogenesis has led to a pharmacopeia of drug manipulations, highlighting the complex interweaving of multiple systems including trophic support, proinflammatory factors, and various neuromodulatory systems (Macht et al., 2020a). Of the neuromodulatory systems which influence neurogenesis, cholinergic regulation of hippocampal neurogenesis is unique in that cholinergic receptors not only directly regulate proliferation of neuroprogenitor cells (Kotani et al., 2006, 2008), but the cholinergic system also indirectly modulates the hippocampal environmental milieu through anti-inflammatory feedback actions (Conejero-Goldberg et al., 2008; Rosas-Ballina and Tracey, 2009; Gnatek et al., 2012; Li L. et al., 2019) and mediation of glial-derived trophic support (Blondel et al., 2000; Wu et al., 2004; Takarada et al., 2012; Pöyhönen et al., 2019). As such, co-disruption of the basal forebrain cholinergic system and neuroimmune signaling pathways can have cascading repercussions on neural health and cognitive function, leading to a predisposition toward neurological disorders that manifest across development and aging.

**FIGURE 1:**
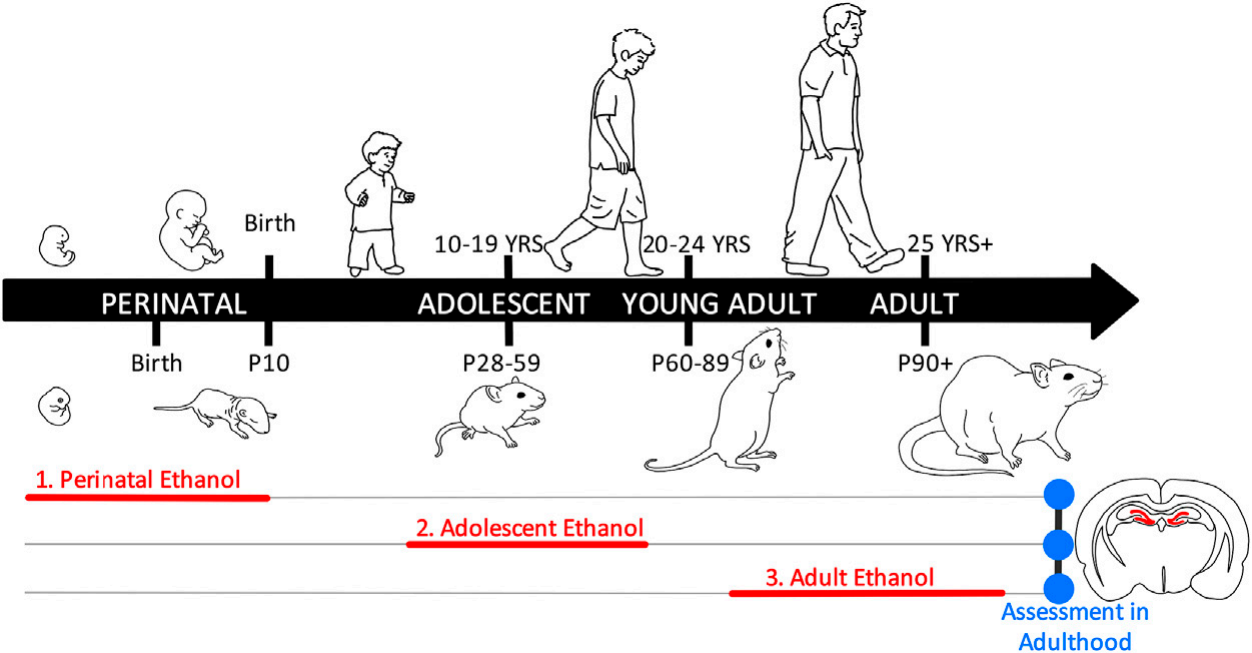
Neurodevelopmental consequences of alcohol exposure on the adult hippocampus. This review aims to compare the long-term effects of perinatal, adolescent, and adult ethanol exposure on adult hippocampal neurogenesis. The developmental impact of ethanol on the basal forebrain cholinergic system and cholinergic signaling within the adult hippocampus will be further discussed in the context of modulation of neuroinflammatory signaling as well as mediation of hippocampal neuroprogenitors and neurogenesis Section 1. The first section will discuss how perinatal ethanol exposure produces maturational changes throughout adolescence and into adulthood, resulting in long-term disruptions in adult hippocampal neurogenesis. Rodent perinatal ethanol exposure models the teratogenic effects of ethanol *in utero* in the human, which often results in a diagnosis of fetal alcohol spectrum disorders (FASD). Of note, the human third trimester, which is the brain growth spurt, corresponds neurodevelopmentally with the first 10 postnatal days (P) in the rat. Therefore, rodent models of human prenatal development must encompass both the prenatal period as well as early neonatal development, collectively termed *perinatal* exposure. It is important to note that due to methodological considerations, the vast majority of rodent models of FASD use either a prenatal or a postnatal design due to confounds in maternal behavior with ethanol-exposed dams Section 2. The second section will discuss the impact of adolescent ethanol exposure on adult hippocampal neurogenesis. Adolescent development is defined in rodents and humans by a collective set of behavioral, cognitive, and physiological characteristics which do not have concrete endpoints. As such, while there is some variation in the cut-off range for this period, traditionally this has been defined from human ages 10–19 years and rodent ages P28-P59, although some human researchers consider adolescent development to continue until through 24 years Section 3. The third section will discuss the impact of adult ethanol exposure on adult hippocampal neurogenesis. In humans, young adulthood (years 20–24) corresponds with rodent P60-P89. Adulthood, which is typically considered at least 25 years of age, corresponds with approximately P90 in the rat. A central theme of this review is that the long-term effects of ethanol exposure depend on the developmental events occurring during ethanol exposure. Therefore, while the age of ethanol exposure will vary by section, all sections will focus on the long-term effects of ethanol with endpoints in adulthood.

In this review, we will focus on a common disruptor of both the basal forebrain cholinergic system and hippocampal neurogenesis: ethyl alcohol (ethanol). Ethanol exposure exerts adverse consequences on the brain throughout the lifespan, with deficits evident from exposure during perinatal development through adulthood. The spectrum of ethanol’s adverse effects produces enormous individual, interpersonal, and societal costs. For example, the robust teratogenic effects of ethanol exposure *in utero* coupled with high rates of ethanol intake in pregnant women (30.3% drink alcohol at some point during pregnancy; 8.3% binge drink at some point during pregnancy) (Ethen et al., 2009) makes fetal alcohol spectrum disorder (FASD) the most preventable neurological disorder in the Western world (Clarke and Gibbard, 2003). Of note, these estimates on drinking during pregnancy can vary slightly across studies depending on inclusion criteria, type of assessment, and whether estimates are assessing changes over time within a population or comparisons between populations. Similarly, ethanol is one of the first and most prevalent drugs to be used and misused by youth (Patrick and Schulenberg, 2014). Furthermore, adolescents are also more likely than any other age group to engage in binge (4–5+ drinks in 2 h) and high-intensity (10+ drinks/session) drinking patterns, which result in high blood ethanol concentrations (BECs) (Chung et al., 2018; Patrick and Terry-McElrath, 2019). The prevalence and adverse effects of ethanol intake continue into adulthood where ethanol use and misuse is associated with even more fatalities than the ongoing opioid epidemic (Esser et al., 2020; Spencer et al., 2020; Mattson, 2021). However, the long-term effects of ethanol exposure on adult hippocampal neurogenesis, cholinergic function, and subsequently on adult cognitive function are highly dependent on the developmental window of exposure. In this review, we will examine the interaction of ethanol exposure across distinct perinatal, adolescent, and adult developmental windows on the maturing cholinergic neurotransmitter and neuroimmune signaling systems in relation to alterations in adult hippocampal neurogenesis (*see* Figure 1).

Each section of this review will discuss the adult impact of ethanol exposure on these systems during discrete neurodevelopmental windows: perinatal, adolescence, and adulthood. Incubation effects, persistence, and reversibility will be discussed, with sex differences highlighted when applicable. The goal of this review is to highlight the molecular underpinnings of ethanol’s impact on hippocampal neurogenesis across the continuum of the lifespan.

## Fetal Alcohol Spectrum Disorder (FASD) and the Lasting Developmental Consequences in Adulthood

Teratogenic disruptions during perinatal (i.e., surrounding birth, which includes *in utero* and neonatal) development induce long-lasting neurodevelopmental consequences on neurological structure and function due to alterations in development of neurocircuitry and other neurobiological systems, permanently shifting maturational trajectories in both the brain and body. One of the most ubiquitously known teratogens is ethanol, and the consequences of ethanol exposure during perinatal development are devastating. These consequences were first documented in 1968 by Paul Lemoine (Lemoine, 1968) in France and later in 1973 by Jones and Smith (Jones and Smith, 1973) in the United States. Since then, clinical and preclinical studies have identified a range of physical, physiological, and behavioral alterations induced by ethanol during perinatal development that are collectively termed FASD; these include physical alterations (e.g., facial dysmorphology, microcephaly, low birth weight) as well as cognitive-behavioral deficits (e.g., hyperactivity, attentional deficits, psychosocial deficits, impaired executive function, learning difficulties, and impaired memory, etc.) (Clarke and Gibbard, 2003). Somewhat surprisingly, despite the global identification and dissemination of information about alcohol’s teratogenic effects to physicians, scientists, and the public, alcohol use and misuse during pregnancy has remained relatively unchanged through the decades (Bhuvaneswar et al., 2007). In fact, alcohol is not only the most commonly used drug by females of reproductive age (24.4% prevalence), but Kanny et al. (2013) found that approximately 12.5% of women continue to drink at binge levels (4+ drinks in 2 h) during pregnancy, resulting in high BECs (0.08% or greater) in relatively short periods of time (Kanny et al., 2013). Investigations into this continuance of alcohol use by women both during pregnancy and while breastfeeding despite alcohol’s known teratogenicity have revealed a complex interplay of factors influencing this ongoing prevalence in behavior, including but not limited to societal pressure, internal and external stress, alcohol dependence, poor understanding of scientific data on substance use during pregnancy, and even inaccurate advice from medical practitioners (Latuskie et al., 2019; Hernandez et al., 2021; Popova et al., 2021). These findings highlight the importance of greater dissemination scientific data on alcohol’s teratogenicity to lay populations and medical practitioners. Alcohol use and binge drinking cause devastating effects to fetal development, resulting in early miscarriage, stillbirth, or upon survival, physical, neurocognitive, and behavioral deficits that are progressively defined along the FASD spectrum, with the most severe deficits resulting in a diagnosis of fetal alcohol syndrome (FAS) (Henderson et al., 2007). Despite these regrettable facts, alcohol use during pregnancy remains the most preventable source of neurological deficits in the Western world (Abel and Sokol, 1987) with estimates of up to 4.8% of school-age children in the United States exhibiting some characteristics of FASD (May et al., 2014).

This section will focus on findings from rodent models of FASD. More specifically, this section will focus on the long-term impact of perinatal ethanol exposure on adult hippocampal neurogenesis, cholinergic function, neuroimmune signaling, and the relationship between these alterations and cognitive-behavioral deficits.

### Perinatal Ethanol Disrupts Hippocampal Neurodevelopment and Adult Neurogenesis: Insight From Rodent Models of FASD

**FIGURE 2:**
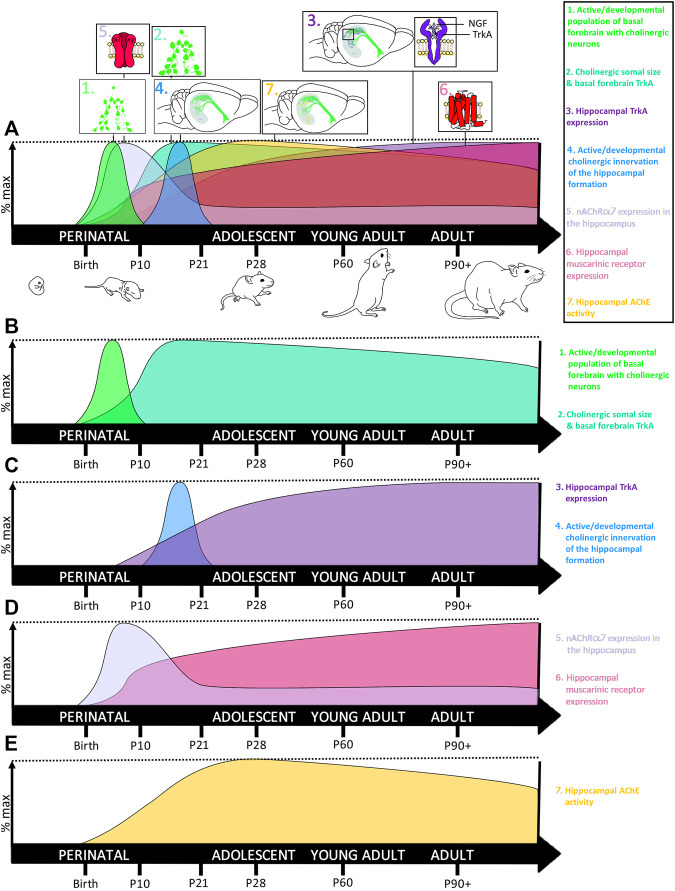
Development of the basal forebrain-hippocampal cholinergic system. **(A)** The cholinergic system undergoes several waves of rapid neurodevelopment during early neonatal and early preadolescent developmental windows, with changes to the hippocampus often lagging behind the basal forebrain. **(B) 1.** Newly differentiated cholinergic neurons aggregate to form the basal forebrain in early neonatal development, with the most rapid increase in ChAT+IR evidenced around the rat postnatal day 7 (Li et al., 1995), during the brain’s growth spurt. **2.** Somal size of these newborn basal forebrain cholinergic neurons continues to increase until weaning, with somal size decreasing slightly into adulthood. **(C) 3.** Somal size is tightly coupled to local increases in NGF binding to TrkA receptors, signaling both cellular differentiation and survival TrkA receptors peak in the medial septum at approximately 21 days and then remain high throughout adulthood. In contrast, TrkA receptors in the hippocampus remain low during neonatal development, begin increasing across adolescence, where they do not reach maximal levels until adulthood (Li et al., 1995). **4.** Rising increases in hippocampal TrkA coincide with onset of basal forebrain innervation of the hippocampal formation, which occurs most robustly between P10-21 and peaks around P17 (Matthews et al., 1974; Nadler et al., 1974). **(D) 5.** Interestingly, nicotinic alpha-7 receptor (nAChR-α7) expression in the hippocampus rises rapidly during early neonatal development, after which hippocampal nAChR-α7 declines, stabilizing to adult levels by preadolescence (Ben-Barak and Dudai, 1979; Court et al., 1997). These reductions in hippocampal nAChR-α7 are thought to parallel developmental periods of synaptic pruning. **6.** In contrast, muscarinic receptor expression in the hippocampus goes through a brief acceleration around P7, and then slowly increases throughout adulthood (Ben-Barak and Dudai, 1979; Court et al., 1997). **(E) 7.** Cholinergic activity within the hippocampus becomes more tightly regulated during adolescence where its extracellular enzymatic degradation by acetylcholinesterase (AChE) peaks during early adolescence, around P30. Thus collectively, the basal forebrain cholinergic system reaches peak maturity during the neonatal period of the brain’s growth spurt, but the cholinergic innervation and regulation of the hippocampus matures during pre- and early adolescence. This early critical window of cholinergic system neurodevelopment makes it sensitive to developmental insults, including ethanol.

The vast majority of newborn neurons are generated in the mammalian brain during perinatal development (Bayer, 1989). This rapid rate of neurogenesis in humans results from an astounding rate of cell division, with approximately 250,000 nerve cells formed every minute and over 80 billion neurons formed in a newborn human, 70 million in a newborn mouse, and 200 million in a newborn rat brain (Bandeira et al., 2009; von Bartheld et al., 2016). Cortical neurons populate their respective regions first, whereas dentate granule neurons of the hippocampus develop later, beginning *in utero* in humans during the third trimester and in the rodent at birth (Bayer, 1980a; 1980b). Concurrently, the hippocampus also begins to express nicotinic cholinergic receptors that regulate hippocampal neurogenesis and regional neurodevelopment (Naeff et al., 1992; Zhang et al., 1998; Adams et al., 2002). Thus, the human third trimester and the first 10 days of rodent postnatal development are equivalent, and constitute the developmental timeframe known as the brain’s growth spurt, which is characterized by rapid hippocampal maturation and the beginnings of hippocampal cholinergic innervation (Dobbing and Sands, 1979; Matthews et al., 1974; Nadler et al., 1974) (*see* Figure 2). In fact, unilateral hippocampal ablation during this early postnatal developmental window reduces cholinergic immunoreactivity in the basal forebrain by 40% in adulthood, highlighting the importance of the reciprocal feedback between the hippocampus and basal forebrain during perinatal neurodevelopment (Plaschke et al., 1997). This reciprocal development between the hippocampus and basal forebrain-to-hippocampus cholinergic projections is dependent upon the production and release of nerve growth factor (NGF), which is selectively produced and released by cells targeted by these cholinergic projections (Higgins et al., 1989). NGF binds to tropomyosin receptor kinase A (TrkA) receptors on cholinergic terminals, providing critical signaling feedback that regulates gene expression necessary for cellular differentiation, influencing somal size, and neurite outgrowth and arborization (as reviewed by (Niewiadomska et al., 2011)), thereby providing continual reciprocal maintenance between cholinergic projection neurons with their target regions during development (Li et al., 1995).

The correlation of human third trimester to the first 10 postnatal days of rodent neurodevelopment (Dobbing and Sands, 1979) has engendered technical complications for the development of FASD rodent models with variations in patterns of ethanol administration (e.g., acute, repeated), developmental period of ethanol exposure (e.g., prenatal, neonatal, or perinatal), and route of ethanol administration (e.g., vapor, intragastric intubation, intraperitoneal injection) (for review *see* (Patten et al., 2014)). In addition, models of FASD must contend with the complications associated with untangling neurological and behavioral outcomes driven by ethanol itself versus alterations in maternal behavior in ethanol-exposed dams, variation in maternal-pup dynamics in ethanol-exposed pups in neonatal split litter models, and other considerations that arise with cross-fostering and artificial rearing. As such, the vast majority of rodent models of FASD use either prenatal (human first and second trimester equivalent) or neonatal (colloquially termed “third-trimester” models) with few studies utilizing models that encompass the entire human three trimesters of pregnancy due to the aforementioned complications. Thus, for the purpose of this review, rodent models of FASD will be referred to generally as perinatal exposure paradigms unless otherwise stated.

The myriad of developmental and pharmacological variations in rodent FASD models complicates translation of findings to the human literature, particularly when results vary by experimental design. For example, the characteristic facial dysmorphology evidenced in children with FASD (i.e., microcephaly, smooth philtrum, small eyes, short nose with a low nasal bridge, and thin upper vermilion) has been specifically linked to ethanol-induced cell death during early embryonic exposure (mouse embryonic days 7–9) (Sulik, 1984) are not evident in rodent neonatal exposure studies that model the human third trimester. These findings highlight alcohol’s unique pathogenic constellation of teratogenic effects that are often dependent on the developmental window of alcohol exposure. However, some consistencies have emerged in the field that are strengthened by their reliability across models despite these technical nuances. For example, reductions in hippocampal volume and cell number as well as deficits on hippocampal-dependent cognitive-behavioral tasks are consistent across various preclinical models of FASD (West et al., 1986; Tran and Kelly, 2003; Gil-Mohapel et al., 2014) and also corroborate findings from humans studies on FASD (Willoughby et al., 2008). This suggests that the hippocampus and related cognitive behavioral outcomes are especially sensitive to the teratogenic effects of ethanol.

#### Assessing Neurogenesis and Limitations From the Current Preclinical Literature on FASD

Some of the molecular deficits induced by perinatal ethanol persist long after the cessation of exposure whereas other deficits only emerge later under system challenges, suggesting that perinatal ethanol exposure can induce latent neurological deficits which manifest over time. For example, while hippocampal cell death and neuronal loss is a common immediate finding in perinatal ethanol exposure models (for review *see* (Gil-Mohapel et al., 2010)), findings regarding the lasting impact of perinatal ethanol exposure on hippocampal neurogenesis in adolescence and adulthood have yielded mixed results. The vast majority of long-term studies on the effects of perinatal ethanol on adult hippocampal neurogenesis have been performed using the thymidine analog 5-bromo-2′-deoxyuridine (BrdU), which is incorporated into dividing cells during the S-phase of mitosis to permanently label newly synthesized DNA, allowing assessments of either proliferation or survival of those cells depending on timing of euthanasia (for a review of findings, *see* Table 1). Although a large percentage of these BrdU+ cells (∼90%) become neurons, a population of these cells also become glia (Nixon and Crews, 2002). As such, BrdU studies in the absence of secondary neuronal markers, such as neuronal nuclei (NeuN) or doublecortin (DCX), the microtubule-associated protein marker of immature neurons, cannot definitively differentiate between neurogenesis and gliogenesis (Nixon and Crews, 2002). The lack of secondary confirmation that changes in BrdU+ cells in adulthood after perinatal ethanol exposure reflect persistent changes in adult hippocampal neurogenesis rather than gliogenesis remains a limitation of the field that needs to be addressed in future studies.

**TABLE 1:** Impact of developmental ethanol exposure on adult hippocampal neurogenesis.

Perinatal ethanol exposure									
Alcohol exposure	Species	Age at assessment	Sex	Hippocampal cell proliferation		Hippocampal cell survival	Neurogenesis	Citation	
*Prenatal Model:* Liquid diet	Rat	BrdU on ∼P30 then 24 h and 1 week until euthanasia	Not specified	↓ BrdU Wheel running reversed deficit		↓ BrdU in ET sedentary rats Wheel running reversed deficit	N/A	Redila et al. (2006)	
*Postnatal Model:* P4-9	Rat	BrdU P32-42; euthanasia P42 and 72	Not specified	− BrdU		-- BrdU in ET sedentary rats ↓ in ET rats relative to CON when stimulated by wheel running	-- BrdU/DCX colocalization in standard housing ↓ BrdU/DCX colocalization in ET rats relative to CON when both groups stimulated by wheel running	Helfer et al. (2009)	
*Three-Trimester Model:* Prenatal and P1-10	Rat	BrdU on P60; euthanasia 2 h or 4 weeks later	Female	-- BrdU -- Ki67 * Wheel running increased in all groups, regardless of ethanol exposure		-- BrdU in ET rats Wheel running increased in all groups, regardless of ethanol exposure	N/A	Boehme et al. (2011)	
*Single postnatal exposure:* P7	Mouse	BrdU daily P7-14; euthanasia P54 P14, P30, P90	Male, Female	N/A		-- BrdU/NeuN in ET rats	N/A	Wozniak et al. (2004)	
*Prenatal Model:* voluntary drinking	Mouse	BrdU ∼P90-102; euthanasia 24 h or 4 weeks later	Male, Female	-- BrdU -- Ki67		-- BrdU in ET rats in standard-housing ↓ in ET rats in enriched environment relative to enriched controls (in both sexes)	↓ BrdU/NeuN colocalization in ET rats relative to CON when both groups stimulated by wheel running	Choi et al. (2005)	
*Prenatal Model:* Liquid Diet	Rat	1 month; 13 months	Male, Female	-- Ki67 (1 month) -- Ki67 (13 months)		N/A	-- DCX (1 month) in ET rats -- DCX (13 months, males) in ET rats ↓ ET females relative to controls (13 months)	Gil-Mohapel et al. (2014)	
*Postnatal Model:* P4-9	Rat	BrdU P41; euthanasia on P72	Male	-- Ki67		↓ BrdU in ET rats Wheel running and complex environments increased BrdU in ET rats	↓ BrdU/NeuN and BrdU/GFAP colocalization in ET rats Wheel running and complex environments increased BrdU/NeuN and BrdU/GFAP in ET rats	Hamilton et al. (2014)	
*Single postnatal exposure:* P7	Mouse	P80	Male, Female	↑ Ki67 (males) -- Ki67 (females)		N/A	↑ DCX (males) -- DCX (females)	Coleman et al. (2012)	
*Single postnatal exposure:* P7	Mouse	P147		↓ BrdU in ET mice ↓ PCNA in ET mice			↓ DCX in ET mice	Ieraci and Herrera, (2007)	
Adolescent ethanol Exposure									
*Self-administration*	Macaque primate	Adult (∼5.5–6.5 years)	Male	↓ Ki67	-- CC3, ↑ FJB		↓ PSA-NCAM	Taffe et al. (2010)	
*Self-administration*	Rat	BrdU P25-27; euthanasia on P44	Male	N/A	↓ BrdU		↓ DCX	Briones and Woods, (2013)	
*Vapor administration*	Rat	P72 and P114	Male	↓ Ki67 -- Ki67	↑ CC3, ↑ FJB ↑ CC3		↓ DCX ↓ DCX	Ehlers et al. (2013)	
*Intermittent intraperitoneal administration*	Rat	P92	Male	↓ Ki67	N/A		↓ DCX	Sakharkar et al. (2016)	
*Intragastric administration*	Rat	P74	Male	-- Ki67	↑ CC3		↓ DCX	Broadwater et al. (2014)	
*Intermittent intragastric administration*	Rat	P56, P80	Male	P56: ↓ Ki67, ↓ Nestin P80: ↓ Ki67, ↓ Nestin *wheel running or indomethacin prevented	P56: ↑ CC3 P80: ↑ CC3 *wheel running or indomethacin prevented		P56: ↓ DCX P80: ↓ DCX *wheel running or indomethacin prevented	Vetreno et al. (2018)	
*Intermittent intragastric administration*	Rat	P56, P220	Male	↓ Ki67	↑ CC3		↓ DCX (P56 - P220)	Vetreno and Crews, (2015)	
*Intermittent intragastric administration*	Rat	P57 or P95 with BrdU +2 h	Male	P57: -- Ki67, -- BrdU, ↓ Sox2, ↓ Tbr2 P95: ↓ Ki67, ↓ BrdU, -- Sox2, -- Tbr2	P57: ↑ CC3 P95: ↑ CC3		P57: -- DCX P95: ↓ DCX, ↓ BrdU/NeuN	Liu and Crews, (2017)	
*Intermittent intragastric administration*	Rat	P70/P73	Male	-- Ki67 ↓ PCNA	↑ CC3/DCX *galantamine prevented/reversed		↓ DCX *galantamine prevented/reversed	Macht et al. (2021)	
*Intragastric administration every 8 h for 4* *days*	Rat	BrdU P38, P40, P45, or P52 with euthanasia +2 h; BrdU P45 with euthanasia P73	Male	-- BrdU P38, 52 ↑ BrdU P45 ↑ Ki67 P45	N/A		↑ DCX P52 ↑ BrdU P52 ↑ BrdU/NeuN P52	McClain et al. (2014)	
*Adult ethanol Exposure*									
*Intragastric administration every 48 h (P70-90)*	Rat	P116	Male	N/A	N/A		-- DCX	Broadwater et al. (2014)	
*(Acute) Single intragastric administration (Chronic) Intragastric administration every 8 h for 4* *days*	Rat	Adult, not specified; (Acute) BrdU post-ethanol with euthanasia 5 or 28 days later; (Chronic) BrdU daily with euthanasia immediately after last ethanol dose or 28 days later	Male	↓ BrdU (acute/chronic) immediately after ethanol	↓ BrdU (chronic) 28 days after ethanol		N/A	Nixon and Crews, (2002)	
*Liquid diet (2* *weeks)*	Rat	>P60; BrdU with euthanasia after last ethanol dose	Male, Female	↓ BrdU (male/female)	N/A		N/A	Anderson et al. (2012)	
*Self-administration (3* *weeks, nondependent); Self-administration plus vapor (+9* *weeks, dependent)*	Rat	Adult, not specified; BrdU 2–4 h after last ethanol exposure with euthanasia 28 days later	Male	↓ Ki67 in non-dependent and dependent ET-rats	↓ BrdU in non-dependent and dependent ET-rats ↑ Fluoro-Jade C in dependent but not non-dependent rats		↓ DCX in non-dependent and dependent ET-rats	Richardson et al. (2009)	
*Intragastric administration every 8 h for 4* *days*	Rat	Adult, not specified; BrdU 4 h before last ethanol dose, and then 3, 7, 14, and 28 days later with euthanasia; BrdU 7 days post ethanol with euthanasia 28–35 days later	Male	↓ BrdU immdiately after last dose; ↑ BrdU on day 7, -- BrdU any other timepoint -- Ki67 (day 7)	N/A		↑ DCX day 14 ↑ BrdU/NeuN day 35	Nixon and Crews, (2004)	
*Self-administration (28* *days) with 14* *days abstinence*	Mouse	∼P105; BrdU 3 days prior to ethanol	Male	↓ PCNA at 14 days abstinence *reversed by desipramine	-- BrdU		↓ DCX at 14 days abstinence *reversed by desipramine	Stevenson et al. (2009)	
*Intragastric administration every 8 h for 4* *days*	Rats	>9 weeks, perfused 8 h after last ethanol dose	Male, Female	↓ Ki67 (male, female)	↑ Fluoro-Jade-B (male, female)		N/A	Maynard et al. (2018)	
*Once-weekly intragastric administration for 11* *weeks*	Rats	Adult, not specified	Female	N/A	N/A		↑ DCX but ↓ total granule neurons	West et al. (2019)	

*N/A, not assessed; ↓, decreased; ↑, increased; --, no change.

#### Perinatal Ethanol Exposure Induces Long-Term Disruptions of Hippocampal Neuroprogenitor Cell Survival

Human studies of FASD indicate prior alcohol exposure results in attenuated age-related increases in hippocampal volume across adolescent development (Willoughby et al., 2008). However, few preclinical studies have reported that perinatal ethanol exposure itself causes persistent reductions of BrdU+ cell proliferation or survival (Redila et al., 2006; Ieraci and Herrera, 2007; Hamilton et al., 2014). For example, rat models of FASD using prenatal ethanol exposure, a three-trimester liquid diet exposure, or a third-trimester neonatal binge exposure have reported no changes in BrdU+ cell proliferation or survival when examined during either adolescence or adulthood under standard housing conditions (Choi et al., 2005; Helfer et al., 2009; Boehme et al., 2011; Gil-Mohapel et al., 2014). Some of these studies did examine co-localization of BrdU+ cells with NeuN and/or examined DCX. In contrast, many of the FASD rodent studies that did not find impaired cell proliferation and/or survival under normal housing conditions reported impairments in cell survival specifically within the hippocampal neurogenic niche when unmasked under positive regulators, including environmental enrichment and exercise, or negative regulators, such as aging (Gil-Mohapel et al., 2014). Several of these studies supporting an unmasking of adult neurogenic deficits after perinatal ethanol exposure similarly report that this deficit was specifically related to reduced cell survival of newborn neurons using co-localization of BrdU with DCX and/or NeuN (Choi et al., 2005; Helfer et al., 2009; Boehme et al., 2011; Gil-Mohapel et al., 2014). This suggests that perinatal ethanol exposure sensitizes the adult neurogenic niche to insults, where deficits become more robust under conditions that challenge neurogenesis either acutely or across the lifespan. Moreover, these studies indicate that unmasking of the reductions in hippocampal neurogenesis after perinatal ethanol exposure is associated with impaired progenitor survival rather than reductions in the proliferating neuroprogenitor pool, suggesting that perinatal ethanol induces subtle long-term disruptions to the hippocampal neurogenic milieu to persistently impact cell death cascades in newborn neurons (*see* Table 1).

Future studies will need to elaborate on these findings, particularly in relation to perinatal ethanol-driven effects on adult hippocampal neurogenesis versus gliogenesis as well as potential sensitivity differences in males versus females. Examination and reporting of both sexes is particularly critical, as some studies have found differences solely in one sex but not the other (*see* Table 1). For example, females seem to be particularly sensitive to age-related unmasking of neurogenic deficits after prenatal ethanol liquid diet exposure, as 13-month-old females but not males exhibited emergent deficits in DCX immunoreactivity that were not evident in early adolescence (Gil-Mohapel et al., 2014). In addition, some variation in outcomes may be due to developmental differences in ethanol’s impact on other concurrently developing neural systems, such as the cholinergic system. For example, differences in perinatal ethanol-induced disruption of hippocampal neurogenesis may shift across adolescence into adulthood when hippocampal innervation becomes refined, or become further exasperated with age as cholinergic systems exhibit age-related neurodegeneration (Schliebs and Arendt, 2011). As such, divergence in outcomes could be partially attributable to differences in prenatal versus postnatal ethanol exposure on brain development, suggesting that more studies encompassing the entirety of the spectrum of human development are critical to reconciling divergent findings due to variations in prenatal versus postnatal exposure on adult hippocampal neurogenesis. Only one study to date has examined the molecular consequences of perinatal ethanol exposure on adult neurogenesis using a three-trimester model (Boehme et al., 2011) – this study examined BrdU in adulthood selectively in females and found that a three-trimester model of FASD did not impact BrdU+ immunoreactivity in adult females (P60), and wheel running increased BrdU+ immunoreactivity across all ages. As it is unclear whether this finding is specific for this age, sex, or glial versus neurogenesis, more studies, and in particular studies that use a three-trimester model, are necessary to clarify these discrepancies.

### Perinatal Ethanol Exposure Alters Maturation of Central Cholinergic Systems: Impact on Adult Neuroinflammatory Signaling and Hippocampal Neurogenesis

The cholinergic system rapidly develops during the early neonatal period in rodents (*see* Figure 2), and is a key regulator of both hippocampal neuroimmune signaling and neurogenesis (Kaneko et al., 2006; Field et al., 2012; Terrando et al., 2014), suggesting that disruptions in maturation of the cholinergic system may have cascading consequences on adult hippocampal neurogenesis in models of FASD. Perinatal ethanol exposure disrupts basal forebrain cholinergic system development, as a single ethanol exposure on postnatal day (P)7 was reported to decrease basal forebrain cholinergic neurons in adulthood (P70) by 34–42% in both male and female rats, relative to age-matched controls (Smiley et al., 2021).

Perinatal ethanol exposure also induces large-scale induction of apoptotic cascades within 24 h after acute ethanol, with variations in regional sensitivity to induction of cell death cascades corresponding with variations in developmental timing of various brain regions (Ikonomidou et al., 2000). For example, peak ethanol-induced induction of the apoptotic marker cleaved caspase-3 in the basal forebrain is most prominent on P7, corresponding with development of the basal forebrain system (Ikonomidou et al., 2000; Olney et al., 2002; Young et al., 2005; Farber et al., 2010). The persistent loss of basal forebrain cholinergic neurons after neonatal ethanol exposure suggests that *1*) ethanol-induced decreases in adult basal forebrain cholinergic neurons may result from caspase-3-mediated apoptotic cell death during critical neonatal developmental windows, and *2*) reductions in adult populations of basal forebrain cholinergic neurons after neonatal ethanol exposure do not recover despite long periods of abstinence. These findings have been reproduced across a variety of mammalian species, from mice to primates, suggesting a high level of congruency in this literature. Moreover, neonatal ethanol-induced loss of basal forebrain cholinergic neurons is accompanied by diminished evoked acetylcholine efflux in the adult hippocampus as assessed using *in vivo* microdialysis, indicating that perinatal ethanol exposure persistently disrupts basal forebrain-hippocampal cholinergic neurocircuitry (Perkins et al., 2015). These findings further highlight that discrepancies in the effects of perinatal ethanol exposure on adult hippocampal neurogenesis may vary across FASD models – particularly prenatal versus postnatal exposure models – as prenatal exposure models miss critical neonatal developmental periods of cholinergic systems, which could subsequently shift the ability of acetylcholine to modulate neuroprogenitors and the hippocampal environmental milieu to regulate adult hippocampal neurogenesis.

#### Perinatal Ethanol Disruptions in Development of the Cholinergic System Subsequently Impair Adult Cognitive-Behavioral Flexibility and Hippocampal Neurogenesis

Loss of basal forebrain cholinergic neurons and subsequent diminution of acetylcholine efflux in the hippocampus have critical implications for cognitive and behavioral deficits in FASD and preclinical models of FASD. For example, deprivation of choline, which is an essential nutrient and critical component of acetylcholine synthesis, exacerbates perinatal ethanol-induced deficits in motor development in early life (Idrus et al., 2017). Conversely, choline supplementation is one of the most researched preclinical interventions in models of FASD and is currently under investigation in clinical trials (Wozniak et al., 2013; Nguyen et al., 2016, Jacobson et al., 2018a; Jacobson et al., 2018b, Wozniak et al., 2020). Clinical evaluation of the efficacy of dietary choline supplementation is supported by a bevy of preclinical research indicating that dietary choline supplementation mitigates cognitive deficits in preclinical models of FASD, particularly on tasks requiring cognitive flexibility. In third-trimester models of FASD, choline supplementation mitigates the long-term effects of neonatal ethanol across both sexes on deficits in discrimination learning (Thomas et al., 2000), spatial working memory (Thomas et al., 2010), spatial reversal learning (Thomas et al., 2004), and trace fear conditioning (Wagner and Hunt, 2006), with findings suggesting that early rodent postnatal choline supplementation is critical (Ryan et al., 2008). A caveat to these findings is that choline is known to exhibit multiple mechanisms of action beyond increasing acetylcholine synthesis, including operating as a methyl donor to regulate epigenetic changes and influencing lipid metabolism due to its role as a precursor for phosphatidylcholine (Bekdash et al., 2013; da Silva et al., 2015; Balaraman et al., 2017). Furthermore, phosphatidylcholine is a primary structural component in both cell and myelin membranes (Sakurai and Kawamura, 1984), making it a critical contributing player to neuronal development. However, choline’s role in acetylcholine synthesis is likely important for its beneficial effects on brain development as cholinesterase inhibitors have revealed convergent beneficial effects in models of FASD. For example, the cholinesterase inhibitor galantamine, which is FDA-approved for the treatment of Alzheimer’s disease (Lilienfeld, 2002; Hampel et al., 2018; Haake et al., 2020), recovered the perinatal ethanol-induced diminution of acetylcholine efflux in the adolescent hippocampus (Perkins et al., 2015). Increasing cholinergic output in the adult hippocampus *via* choline supplementation or cholinesterase inhibition may also mitigate perinatal ethanol-induced deficits in hippocampal neurogenesis, as the cholinesterase inhibitors galantamine and donepezil both increase cholinergic neurotransmission and hippocampal neurogenesis in adult rats (Kotani et al., 2006, Kotani et al., 2008). This suggests that broadly increasing acetylcholine neurotransmission may produce downstream beneficial effects on hippocampal neurogenesis (Kotani et al., 2006, Kotani et al., 2008; Kita et al., 2014; Madrid et al., 2021), further highlighting *1*) the critical role of acetylcholine in neurogenesis, *2*) the likely contribution of acetylcholine reductions to loss of neurogenesis in these model, and *3*) the therapeutic potential of compounds which target the cholinergic system in FASD.

#### Perinatal Ethanol Disrupts Nicotinic and Muscarinic Receptor Expression in the Adult Hippocampus

One of the mechanisms by which perinatal ethanol-induced disruption of basal forebrain-hippocampal cholinergic neurocircuitry may affect adult hippocampal neurogenesis is through long-term alterations in cholinergic receptor activation in adulthood. Both muscarinic and nicotinic receptors directly affect neuroprogenitor pools and indirectly regulate the hippocampal environmental milieu. Muscarinic M1 receptors are abundant in the adult hippocampus, where they colocalize with newborn cells in the dentate gyrus to play a key role in hippocampal cell proliferation (Levey et al., 1995; Mohapel et al., 2005). In fact, M1 receptor activation is sufficient to rescue cell proliferation deficits in a model of basal forebrain cholinergic denervation (Van Kampen and Eckman, 2010). However, there are conflicting findings regarding the impact of perinatal ethanol on adult muscarinic receptor expression, often reflecting a divergence in adult outcomes due to differing developmental periods of ethanol exposure across FASD rodent models. For example, ethanol exposure across prenatal development reduced adult (i.e., P90) hippocampal muscarinic receptor density in male and female rats (Black et al., 1995), whereas ethanol exposure from P4 to P10 using intragastric intubation which resulted in cyclical, high blood ethanol concentrations increased hippocampal muscarinic receptor density and decreased their dissociation constant in adulthood (P90) across both sexes (Kelly et al., 1989). Interestingly, muscarinic receptor dynamics are not affected by dosing regimens that result in consistent BECs. Non-cyclical, stable level BECs were achieved by spreading 12 feedings of 2.5% (v/v) ethanol consistently across a 24-h period, suggesting that muscarinic receptors are impacted not only by developmental window, but also by circulating BECs. These results highlight that both developmental timing and dosing regimen of ethanol exposure can create complex alterations in muscarinic receptor expression and affinity dynamics (Kelly et al., 1989).

Interestingly, the sole study reporting that ethanol exposure across gestation decreases adult cell proliferation (Redila et al., 2006) parallels the finding that ethanol exposure across gestation similarly decreases hippocampal muscarinic receptor density in adulthood (Black et al., 1995). Likewise, FASD models that use the third-trimester neonatal binge paradigm (i.e., P4-P10) typically do not report long-term deficits in hippocampal cell proliferation, with one study reporting increased cell proliferation in adult males after a single ethanol dose on P7 (Coleman et al., 2012). These discrepancies highlight that a full three-trimester model, which more accurately reflects human prenatal development by encompassing both the prenatal and postnatal periods, is critical to reconcile the prenatal versus postnatal literature discrepancies regarding hippocampal muscarinic receptor dynamics, and therefore is an important future direction for the field.

In contrast to muscarinic receptors, nicotinic receptors have received little attention in relation to the lasting effects of perinatal ethanol exposure, with a single study reporting that hippocampal α7 nicotinic receptor density in adulthood is unaffected by perinatal (i.e., P2-P10) ethanol exposure (Perkins et al., 2015). However, reductions of basal forebrain cholinergic tone in the hippocampus may have cascading consequences on nicotinic receptor activation that are similarly important for adult neurogenesis. In fact, choline is also a selective agonist at the α7 nicotinic receptor (Alkondon et al., 1997), and loss of cholinergic activation of the α7 nicotinic receptor could impair important anti-inflammatory feedback onto microglia (Li L. et al., 2019), disrupting the inflammatory balance in the hippocampal environmental milieu. This suggests that a more thorough assessment of perinatal ethanol’s impact on nicotinic receptor dynamics and the long-term mediation of nicotinic signaling the adult neurogenic niche remains an important future direction for the field.

#### Perinatal Ethanol Acutely Disrupts the Proinflammatory-anti-inflammatory Balance in the Neurogenic Niche With Proinflammatory Gene Induction Exacerbated in Adulthood Following Immune Challenges

A growing body of evidence has implicated the innate immune system not only in pathogen defense, but also in neurodevelopment (for review *see* (Macht, 2016)). While microglia are classically associated with the brain’s immune response, it is becoming increasingly appreciated that astrocytes and neurons can also contribute to proinflammatory signaling cascades. However, the acute effect of ethanol on innate immune induction in preclinical models of FASD may depend on the timing of microglial regional increases in population across the CNS. For example, while neurons and astrocytes exhibit common neuroepithelial origins, ontogeny of neurons precedes that of astrocytes, with the vast majority of astrocytes not produced until first postnatal month in the rodent (Qian et al., 2000; Sauvageot and Stiles, 2002). Microglia, in contrast, are derived from developing macrophages in the yolk sac during early embryogenesis (peaking on approximately embryonic day [E]7) and then migrate to the developing nervous system whereupon they continue to proliferate, populating developing brain regions in a caudal-rostral manner (Alliot et al., 1999; Ginhoux et al., 2010). Interestingly, a single 50-min exposure to ethanol in a vapor chamber on gestational day 12.5 increased microglia undergoing the S phase of the cell cycle, which includes critical periods of DNA synthesis (Salem et al., 2021). Future studies would need to examine if these changes in the microglial cell cycle underlie congruent findings from several studies that ethanol also acutely increases proinflammatory gene induction in the developing prenatal and neonatal brain, likely reflecting varying levels of neuroimmune induction within both neurons and glia, including microglia.

Specifically, prenatal ethanol exposure increased CCL6, IL-21, IL-10ra, and TNFα expression in the developing brain of male and female rats, relative to age-matched controls, on E17 (Terasaki and Schwarz, 2016), with female rats exposed to ethanol also exhibiting increases in CCL2, CCL5, CCL9, CXCL10, and IL-5 in whole brain homogenates. Postnatal ethanol exposure on P5 also acutely increases hippocampal proinflammatory gene expression of IL-1β and CCL4 in male and female rats (Ruggiero et al., 2018), with multiple exposures to ethanol (P4-P9) resulting in even more dramatic induction of proinflammatory signaling cascades, including IL-1β, TNFα, CD11b, and CCL4 in both sexes (Boschen et al., 2016). Similarly, a single exposure to ethanol on P4 in mice results in significant increases in CCL2 and monocyte chemotactic protein-induced protein (MCPIP) in the brain and in cultured microglial cell lines over the course of several hours (Zhang et al., 2018). Increased CCL2 signaling after ethanol is particularly important as it regulates acute induction of apoptotic cascades after developmental (P4) ethanol exposure (Zhang et al., 2018), suggesting that ethanol induction of neuroimmune cascades early in development may directly contribute to neuronal loss, including loss of developing cholinergic neurons. Indeed, persistent increases in CCL2 signaling (Pascual et al., 2017) by perinatal ethanol may increase sensitivity of adult newborn hippocampal neurons to cleaved caspase-3-induced apoptosis, reducing their successful integration into hippocampal neurocircuitry.

The effects of perinatal ethanol on innate immune activation are further exacerbated in adulthood. This is in part due to the fact that perinatal ethanol has cascading repercussions on immune regulation of the developing cholinergic system to impact later neuroimmune signaling dynamics in adulthood. For example, some studies suggest perinatal ethanol sensitizes later innate immune gene responses in adulthood, in part due to diminished capacity for cholinergic anti-inflammatory feedback that leads to an exaggerated proinflammatory neuroimmune response. Prenatal ethanol exposure (2 g/kg ethanol, twice daily, E10-E16) dramatically exacerbates the adult response to modest innate immune challenges (25 μg/kg lipopolysaccharide [LPS]), evidenced by exaggerated hippocampal gene expression of IL-1β and IL-6 in male rats but not female rats, relative to controls (Terasaki and Schwarz, 2016). Similar long-term alterations in the peripheral immune system in adulthood (approximately 4 months old rats) have been evidenced after gestational exposure to ethanol vapor across days 8–19, with adult males but not adult females exhibiting increased levels of circulating monocytes (Bake et al., 2021). Conversely, adult males exhibited decreased basal levels of peripherally circulating cytokines whereas adult females exhibited elevated peripheral levels of circulating cytokines. The authors speculate that low circulating peripheral cytokines in adult males under basal conditions could reflect inappropriate immune responsivity to infection, possibly contributing to higher rates of persistent systemic infections in males with FASD. These findings that prenatal ethanol exposure increases sensitivity to neuroimmune signaling induction in adult males, but not females, may have critical implications, as human studies report that males are more likely to receive an early diagnosis of FASD, reflective of increased phenotypic severity (Thanh et al., 2014; DiPietro and Voegtline, 2017; Osborne et al., 2018).

In addition, persistent increases in CCL2 signaling (Pascual et al., 2017) by perinatal ethanol may increase sensitivity of adult newborn hippocampal neurons to cleaved caspase-3-induced apoptotic cascades, reducing their successful integration into hippocampal circuitry. These persistent proinflammatory effects induced by ethanol appear to be mediated by TLR4 signaling cascades, as TLR4-deficient mice do not exhibit long-term upregulation of several cytokines and chemokines, including IL-1β and CCL2, in models of perinatal ethanol exposure (Pascual et al., 2017). Thus, not only does ethanol’s acute induction of CCL2 and TLR4 signaling pathways during perinatal development contribute to acute increases in proinflammatory gene expression, but these effects also persist into adulthood. Adult exposure to LPS induces a sensitized CCL2 and TLR4 signaling response in the hippocampus in rodent models of FASD, contributing to increased apoptosis in the adult neurogenic niche and greater sensitivity to proinflammatory-induced loss of adult neurogenesis.

**FIGURE 3:**
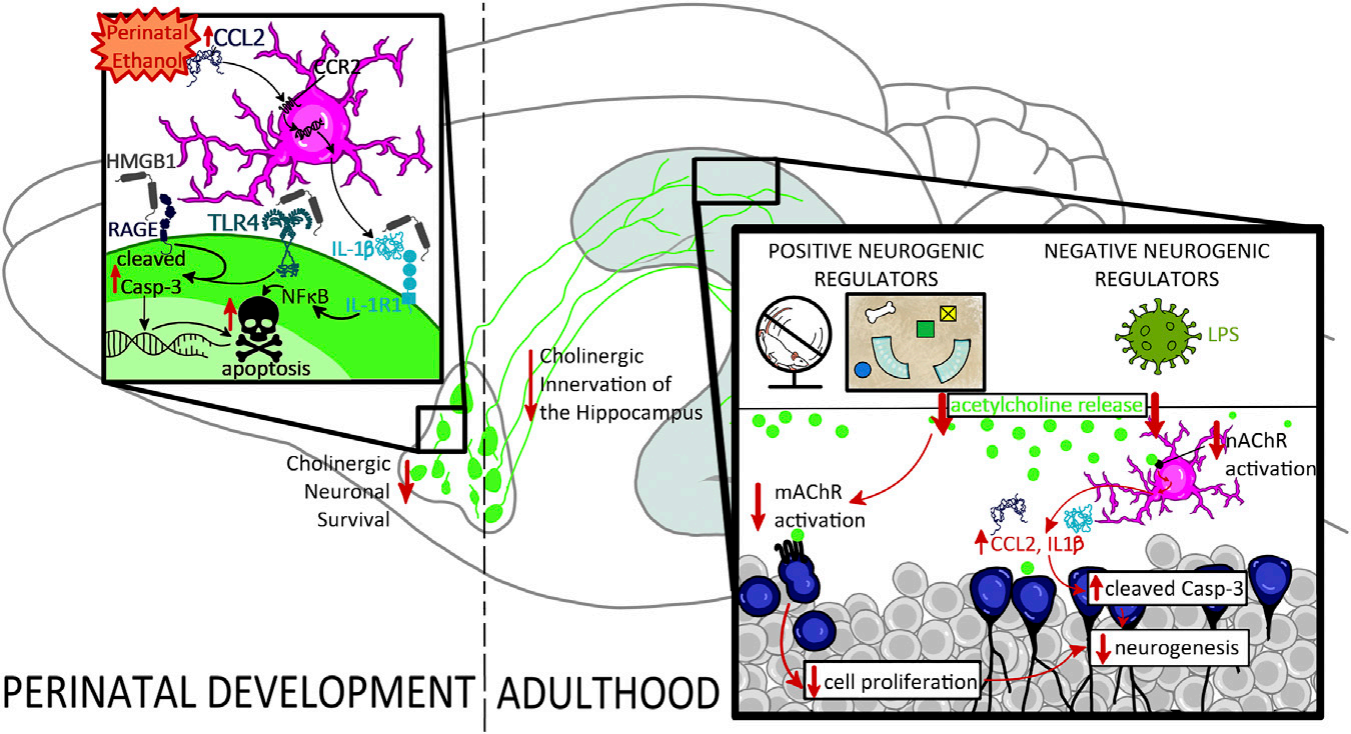
Proposed mechanism underlying the impact of perinatal ethanol on adult hippocampal neurogenesis. Perinatal ethanol acutely increases CCL2 proinflammatory cascades, resulting in increases in IL-1β and HMGB1 gene expression as well as activation of TLR4, RAGE, and IL-1R1 receptor signaling, leading to increased activation of cleaved caspase-3 (Casp-3) pathways, and resulting in cellular apoptosis. Activation of cell death pathways results in basal forebrain cholinergic cell death during critical developmental periods. This persistent ethanol-induced loss of basal forebrain cholinergic neurons persists into adulthood and results in long-lasting neurodevelopmental repercussions. A long-term consequence of these perinatal ethanol effects is a hypofunctioning cholinergic network evidenced by decreased capacity for stimulated acetylcholine release in the hippocampus in adulthood. Acetylcholine release is stimulated by positive neurogenic regulatory such as voluntary exercise and environmental enrichment, as well as negative neurogenic regulatory factors such as LPS. Loss of hippocampal cholinergic signaling can unmask deficits in adult neurogenesis, decreasing either cell proliferation, or, more frequently, increasing cell death through hypersensitivity to proinflammatory gene induction and activation of Casp-3 apoptotic pathways.

Collectively, these findings indicate that perinatal ethanol-induced acute induction of proinflammatory signaling cascades may be an early underlying factor in developmental cholinergic deficits by contributing to their programmed cell death during rodent neonatal or human third-trimester development. In adulthood, these developmental deficits in cholinergic function may unmask greater impairments under conditions of neuronal stress, potentially through alterations in muscarinic and nicotinic receptor activation and consequential augmented disruption of the proinflammatory signaling in the hippocampal environmental milieu, reflecting a vicious cycle that may drive both impairments in neurogenesis and greater cognitive deficits in high-complexity tasks (*see* Figure 3).

### Restoration and Treatment Challenges in Models of FASD

Children with FASD have several complications due to large-scale cell death induced by prenatal alcohol exposure coupled with broad shifts in epigenetic regulation of gene expression. Epigenetics is the modulation of gene expression in the absence of alterations to DNA, making it a master director of brain development by altering gene accessibility to transcriptional machinery across time (Podobinska et al., 2017). For example, the most frequently investigated epigenetic modification is changes in patterns of methylation across the genome. Increases in DNA methylation on CpG dinucleotides at gene promoter regions restrict transcriptional machinery’s access to specific genes, thereby reducing transcription and translation into protein. In contrast, removal of these methyl groups at CpG-rich promoter islands increases gene access to transcription machinery, resulting in increased gene transcription. Changes in methylation at various gene loci are a critical part of normal neurodevelopment and can be further modified by environmental exposures, including maternal behavior (Weaver et al., 2004) and alcohol exposure (Kobor and Weinberg, 2011), with increased sensitivity across different periods over the lifespan. Epigenetics, including DNA methylation, therefore has the capacity to alter gene expression with tight temporal regulation during critical developmental periods. These neurological and epigenetic changes during critical brain developmental windows shift the brain’s developmental trajectory in children with FASD, resulting in a variety of social, emotional, cognitive, and behavioral deficits that emerge with age (Kodituwakku, 2009). This suggests several considerations in the context of FASD: *1*) ethanol-induced alterations during perinatal development persistently impact development by changing gene transcription, *2*) some consequences of perinatal ethanol will emerge over time in conjunction with developmental changes in epigenetic regulation of gene expression, and *3*) pharmacological interventions which aim to reverse perinatal ethanol-induced developmental effects will be most effective if they also reverse perinatal ethanol-induced epigenetic changes.

The emergence of ethanol’s cascading consequences on neurodevelopment over time complicates interventions aimed at reversal of neurological and behavioral deficits in models of FASD. In fact, the majority of current pharmacological intervention strategies approved for children with FASD target other neuropsychiatric comorbidities, such as the use of stimulants for the highly co-morbid diagnosis of attention deficit hyperactivity disorder in FASD children, with few treatment strategies aimed to prevent or reverse FASD pathophysiology itself (Martinez and Egea, 2007; Wozniak et al., 2019). These challenges in specifically treating FASD are highlighted in preclinical findings surrounding the cholinergic system. For example, using a third-trimester model (P4-P9), Milbocker and Klintsova (Milbocker and Klintsova, 2021) reported that ethanol reduced cholinergic neuron populations (identified by choline acetyltransferase (ChAT)) in the young adult basal forebrain (P72). This deficit was not recovered with either voluntary exercise, complex environmental housing, or a combination of the two (P30-72). Interestingly, choline supplementation has yielded some success with preclinical studies, suggesting that prenatal choline supplements given in conjunction with ethanol exposure across gestation can mitigate loss of brain weight in models of FASD (Thomas et al., 2009) as well as prevent adult working memory deficits in the Morris water maze (Thomas et al., 2010). Choline supplementation also attenuates deficits in cholinergic muscarinic receptor binding (Monk et al., 2012) and causes broad changes in brain gene regulation including cholinergic content as well as receptor expression and function (Otero et al., 2012). However, while one mechanism of choline supplementation is through enhanced acetylcholine synthesis, choline supplementation also reverses perinatal ethanol’s increases in DNA methylation in the hippocampus (Otero et al., 2012). The ability of choline supplementation to reverse alternations in hippocampal DNA methylation after perinatal ethanol is an exciting finding, but these interactions are complex as choline supplementation exhibited opposing effects in the absence of ethanol, highlighting that the role of choline supplementation in DNA methylation must be taken in context with expression of methyltransferase activity and other epigenetic regulators. To further complicate the mechanism of choline supplementation as a therapeutic in models of FASD, choline also affects lipid metabolism and enhances liver oxidation of fatty acids. This suggests that choline supplementation may affect hippocampal gene transcription indirectly through broader impacts on the body in general. These results highlight the multifaceted mechanisms by which choline supplementation may help mitigate developmental consequences of ethanol, as choline is a precursor for the synthesis of acetylcholine, a methyl donor, influencing epigenetic regulation of gene transcription (Zeisel, 2006), and a regulator of lipid metabolism. Collectively, this suggests that ethanol and choline supplementation effect gene transcription broadly, and hippocampal gene transcription specifically.

The positive preclinical effects of choline supplementation on FASD behavioral and neurological outcomes have given it a spotlight in clinical trials, several of which are ongoing (Wozniak et al., 2020). However, clinical trials have further highlighted that choline supplementation efficacy relies heavily on a preventative and/or early intervention strategy and has not yielded clinical success when implemented later in life (e.g., in school-age children) (Nguyen et al., 2016), suggesting the effective therapeutic window for choline supplementation is narrow. The narrow therapeutic window in FASD remains an ongoing clinical challenge (Idrus and Thomas, 2011), particularly for intervention strategies aimed at mitigating deficits in females with FASD, as they are often diagnosed later in life than males (DiPietro and Voegtline, 2017; Osborne et al., 2018). Thus, sex differences in FASD presentation and biological alterations are important to consider in the search for novel treatments for FASD in older populations.

### Adolescent Ethanol Exposure and the Developmental Impact in Adulthood

Adolescence is a period of rapid brain maturation with extensive myelination, synaptic pruning, and neurogenesis, highlighting that remodeling of molecular circuitry is a critical component of this developmental period that parallels rapid behavioral and cognitive growth (Arain et al., 2013). Adolescence is also a period when alcohol experimentation and use is typically initiated across both sexes (Petit et al., 2013). As adolescents are less sensitive than adults to the soporific effects of alcohol, they tend to achieve higher BECs than adults in a single drinking session. This means that adolescents are far more likely than adults to consume alcohol in a binge drinking pattern (Kuntsche and Gmel, 2013), which is defined by the National Institute on Alcohol Abuse and Alcoholism (NIAAA) as consuming 4+/5+ drinks in a 2-h period for women and men, respectively (Alcohol Policy Information System, 2020; Drinking Levels Defined, 2021). In fact, the vast majority of alcohol consumption in adolescents is characterized by intake during weekend binge drinking sessions (Chung et al., 2018), where periods of high levels of alcohol intake are intermittently dispersed with short periods of abstinence. The percentage of individuals engaged in binge drinking sessions escalates across adolescence and into college age, with approximately 4% of eighth grade, 9% of 10th grade, 14% of 12th grade, and 44% of college students reporting recent binge drinking episodes (Wechsler et al., 1995; O’Malley et al., 1998; Johnston et al., 2019). Moreover, adolescent-specific neuromaturation persists in humans through age 25 (Crews et al., 2019), well beyond the legal drinking age, suggesting that these persistent neurodevelopmental disruptions which are continuing to be elucidated in clinical and preclinical studies potentially impact a broad spectrum of the legal drinking population.

The long-term consequences of alcohol drinking in adolescence have primarily been examined through two collaborative consortiums: *1*) National Consortium on Alcohol and Neurodevelopment in Adolescence (NCANDA), which evaluates the progressive disruption of neurodevelopment and behavioral alterations with alcohol exposure across adolescence in humans, and *2*) the Neurobiology of Adolescent Drinking in Adulthood (NADIA) consortium, which uses rodent models of adolescent intermittent binge ethanol exposure to examine mechanisms underlying ethanol’s long-term molecular and cognitive-behavioral changes in adulthood (Crews et al., 2019). As adolescents who binge drink often continue drinking in adulthood, discerning the discrete effects of adolescent versus adult alcohol exposure can be complex in human studies. Thus, the NADIA consortium has modeled patterns of human adolescent binge drinking in rodents using a paradigm of adolescent intermittent ethanol (AIE) exposure in rats. In this model, ethanol exposure occurs specifically across periadolescent development (P25-P55) *via* an intermittent dosing regimen that results in the high BECs (>150 mg/dl) that are achieved in human adolescents (Lamminpää et al., 1993; Kraus et al., 2005; White et al., 2006; Donovan, 2009; Patrick et al., 2013; Orio et al., 2018), followed by a period of abstinence to determine the long-lasting, persistent effects of adolescent ethanol exposure on the brain and behavior in adulthood (for a review of the model, *see* (Crews et al., 2019)). Findings from the NADIA consortium and other rodent models of adolescent ethanol exposure have indicated that the effects of ethanol on the brain and behavior during adolescence are distinct from perinatal or even adult exposure, instead being molded from the landscape of adolescent development where neurocircuitry is being remodeled and refined versus formed. The result is a plethora of cognitive-behavioral and neurobiological changes that persist into adulthood despite abstinence (as reviewed by (Crews et al., 2019)). This section will highlight some of these persistent effects, particularly in relation to adult hippocampal neurogenesis, the cholinergic system, and neuroinflammation.

### Vulnerability to Neurogenic Insults During Adolescence and the Long-Term Impacts on the Adult Hippocampus

Neurogenesis during adolescent development is on average four-fold greater than during early adulthood (He and Crews, 2007; Kozareva et al., 2019), and this developmental finding is consistent across mammalian species (Snyder, 2019). High levels of adolescent neurogenesis are thought to reflect a heightened need for neuroplasticity during this period, which corresponds with adolescent-related behavioral changes (Kozareva et al., 2019). Generally, adolescence is characterized by increased exploration, risk-taking behaviors, inhibition of juvenile behavioral patterns, and acquisition of new behaviors essential for transition from parental care to independence as an adult (Spear L. P., 2000). As neurogenesis is an index of neuroplasticity and facilitates pattern separation, spatial learning and memory, and cognitive flexibility, it follows that heightened neurogenesis during adolescence contributes to adaptive behavioral development during this period. However, the increased need for hippocampal neuroplasticity during normal adolescent neurodevelopment also confers a period of neurogenic vulnerability with ensuing consequences on behaviors that rely upon this mechanism of cellular plasticity.

#### Adolescent Binge Ethanol Exposure Impairs Adult Hippocampal Neurogenesis by Disrupting Cell Proliferation and Inducing Cell Death Cascades

Newborn neurons during adolescence are particularly sensitive to ethanol, showing dose-dependent reductions in hippocampal neurogenesis after acute ethanol exposure (Crews et al., 2006). The magnitude of this deficit in hippocampal neurogenesis after acute adolescent ethanol exposure is somewhat extraordinary, as identical amounts of ethanol acutely reduce neurogenesis by 80% in adolescence versus 30% in adulthood when normalized to respective developmental control levels of neurogenesis (Crews et al., 2006). More concerning are the long-term consequences of adolescent binge ethanol exposure. Our laboratory has found that not only does AIE exposure reduce adult expression of DCX, a neuroprogenitor cytoskeleton protein, in late adolescence (24 h post-AIE), but this reduction in DCX expression persists well into adulthood (P220) in both the dorsal and ventral dentate gyrus (Nixon and Crews, 2002; Crews et al., 2006). This finding reproduces across species (mice, rats, and nonhuman primates) as well as across various routes of administration (intragastric intubation, intraperitoneal injection, vapor) (*see* (Macht et al., 2020a) for review), and is unique to this developmental window. For example, as previously discussed, perinatal ethanol exposure only mildly reduces adult hippocampal neurogenesis, often requiring a challenge to unmask deficits. Similarly, chronic but not intermittent ethanol exposure in adulthood reduces neurogenesis in a transient manner which recovers following periods of abstinence (Nixon and Crews, 2002; Broadwater et al., 2014). This is not true following intermittent alcohol exposure across adolescence, where reductions in hippocampal neurogenesis are persistent, lasting well into adulthood, perhaps for the duration of the organism’s life, despite abstinence (Nixon and Crews, 2002; Crews et al., 2006).

The enduring loss of hippocampal newborn neurons after AIE most likely involves both subtle disruptions in the neuroprogenitor cell proliferating pool as well as more robust findings involving decreased survival of neuroprogenitors as they differentiate into neurons and integrate into hippocampal circuitry (for summary *see* Table 1). Cell proliferation after AIE has most frequently been evaluated using staining of the nuclear protein Ki67, which has high fidelity with BrdU expression patterns for proliferation (Kee et al., 2002). Ki67 is expressed during mitosis across mammalian species and exhibits a very short half-life, making it a conservative but accurate estimate of actively dividing cells in the subgranular zone of the neurogenic niche. Some (Nixon and Crews, 2002; Taffe et al., 2010; Vetreno and Crews, 2015; Liu and Crews, 2017) but not all (Ehlers et al., 2013; Broadwater et al., 2014; Macht et al., 2021) studies have found that adolescent binge ethanol exposure decreases Ki67 immunoreactivity in adulthood. Conversely, adolescent binge ethanol exposure has consistently been found to induce activation of the apoptotic executioner caspase cleaved caspase-3 (Ehlers et al., 2013; Broadwater et al., 2014; Vetreno and Crews, 2015; Liu and Crews, 2017; Macht et al., 2021), and/or to increase hippocampal necrotic cell death, as marked by Fluoro-Jade B (Taffe et al., 2010; Ehlers et al., 2013), across species and across variations in route of administration. Moreover, AIE increases expression of cleaved caspase-3 specifically within DCX-labeled neurons, suggesting that activation of cell death machinery directly contributes to loss of newborn hippocampal neurons (Macht et al., 2021). Collectively, these findings suggest that adolescent binge ethanol exposure disrupts the neurogenic niche in ways that interfere with the successful maturation of adult newborn neurons, potentially through mechanisms involving either *1*) a failure to assimilate signals driving successful integration into existing hippocampal circuitry or *2*) hypersensitivity to the stimulation of cell death executioner pathways.

#### Adolescent Binge Ethanol Exposure Impairs Adult Hippocampal Neurogenesis by Disrupting Cell Proliferation and Inducing Cell Death Cascades

One mechanism that activates cell death pathways in adult newborn neurons is induction of proinflammatory signaling cascades, resulting in cleavage of caspase-3 and consequential catalyzation and cleavage of critical cell proteins, chromatin condensation, DNA fragmentation, and ultimately cellular apoptosis (Porter and Jänicke, 1999; Macht et al., 2021). Chronic induction of proinflammatory signaling cascades within the hippocampus are a persistent molecular consequence of adolescent ethanol exposure (Crews et al., 2016), and AIE-induced upregulation of proinflammatory gene expression is evident across multiple signaling steps, including increasing the nuclear histone-binding protein high mobility group box protein 1 (HMGB1) (Swartzwelder et al., 2019; Macht et al., 2021), CCL2 (Macht et al., 2021), Toll-like receptor 4 (TLR4), the canonical neuroimmune gene transcription factor nuclear factor kappa-light-chain-enhancer of activated B cells p65 (pNFκB p65) (Li Q. et al., 2019), and cyclooxygenase-2 (COX-2) (Pascual et al., 2007; Macht et al., 2021).

HMGB1 is a central player in these molecular cascades within the hippocampus, with multiple studies demonstrating AIE increases hippocampal granule cell expression of HMGB1 immunoreactivity in the adult dentate gyrus (Vetreno et al., 2018; Swartzwelder et al., 2019; Macht et al., 2021). Upon stimulation, HMGB1 is translocated from the nucleus to the cytoplasm whereupon it is actively and passively secreted into the extracellular space. Once in the extracellular space, HMGB1 functions are multifaceted as HMGB1 has the potential to act as a solo player wherein it binds to and activates a variety of innate immune receptors, including TLR4 and the receptor for advanced glycation end products (RAGE) (Miyata et al., 1996; Yanai et al., 2009), or it can act in concert with other extracellular factors, forming complexes with other immune molecules including IL-1β to subsequently potentiate their responses (as reviewed by (Bianchi, 2009)). Thus, induction of HMGB1→TLR4/RAGE→pNFκB p65 signaling cascades may be a critical mediator in AIE-induced cell death of neuroprogenitors, as expression of hippocampal pNFκB p65 and cleaved caspase-3 are highly correlated (He and Crews, 2007). Furthermore, reversal of hippocampal proinflammatory cascades through the cholinesterase inhibitor galantamine prevents and reverses both AIE induction of HMGB1, COX-2, and CCL2 as well as reduces AIE induction of cleaved caspase-3 in DCX-labeled immature neurons in male rats (Macht et al., 2021), further highlighting that disruption of cholinergic signaling contributes to AIE induction of neuronal inflammatory proinflammatory cascades, cell death, and adult hippocampal neurogenesis.

Hippocampal microgliogenesis and partial microglial activation are also evident in adulthood after AIE, evidenced by increases in the number of hippocampal Iba+ cells as well as shifts in these Iba1+ cells towards a less ramified morphological state and increases in microglial (Iba-1+immunoreactive) expression of pNF-κB p65 (McClain et al., 2011; Liu and Crews, 2017). This phenotypic shift of hippocampal microglia by adolescent binge ethanol exposure may contribute to the observed increases in expression of several proinflammatory cytokines in the adult hippocampus after adolescent binge ethanol exposure, including IL-1β, TNFα, and IL-6 (Gómez et al., 2018). Consequences of increased extracellular IL-1β are magnified by concurrent increases in HMGB1, as IL-1β and HMGB1 form complexes to increase affinity at TLR4 (Coleman et al., 2018), potentiating proinflammatory signaling. This suggests that neurons and microglia work in concert to potentiate proinflammatory responses and activate apoptotic pathways in newborn hippocampal neurons in adulthood.

### Adolescent Binge Ethanol Suppresses Basal Forebrain Cholinergic Systems to Induce Adult Neuroinflammatory Signaling and Impair Hippocampal Neurogenesis

Adolescent binge ethanol exposure causes a loss of basal forebrain cholinergic neurons immediately following the conclusion of ethanol treatment (i.e., P55) that persists well into adulthood (i.e., P220 [165 days post-ethanol]), paralleling AIE-induced lasting reductions in hippocampal neurogenesis and proinflammatory induction. The observed 20–30% reduction in basal forebrain cholinergic neuron number following adolescent binge ethanol exposure is consistent across both mouse and rat studies (Coleman et al., 2011; Vetreno and Crews, 2018; Macht et al., 2020a; Vetreno et al., 2020; Crews et al., 2021) in both sexes (Vetreno and Crews, 2018). However, unlike in models of FASD, the reduction in cholinergic neuronal markers after adolescent binge ethanol exposure is preventable and reversible with exercise (Vetreno and Crews, 2018) as well as with repeated treatment with the cholinesterase inhibitor galantamine (Crews et al., 2021) and the non-steroidal anti-inflammatory compound indomethacin (Vetreno and Crews, 2018). Restoration of basal forebrain cholinergic neurons after AIE also restores hippocampal neurogenesis, highlighting the reciprocal dynamic between these systems (Macht et al., 2021). These findings suggest not only that the persistent loss of cholinergic anti-inflammatory feedback after AIE is a central mechanism in the long-term induction of hippocampal proinflammatory signaling cascades, but also that proinflammatory signaling plays a role in AIE-induced cholinergic pathology more locally within the basal forebrain, evidenced by AIE increased phosphorylation of NF-κB p65 within cholinergic neurons (Vetreno et al., 2020).

One could assume that loss of expression of ChAT immunoreactivity after AIE – as with findings in fetal alcohol models – suggests cell death of this neuronal population. However, this conclusion from the perinatal literature is fueled by *1*) concurrent induction of caspase activity of these neurons during critical neurodevelopmental windows during the brain’s growth spurt, and *2*) failure to restore ChAT immunoreactivity with pharmacological or environmental interventions (Ikonomidou et al., 2000; Olney et al., 2002; Young et al., 2005; Farber et al., 2010; Smiley et al., 2021). Findings from AIE do not parallel the fetal alcohol field in this regard. For example, there is no loss of total neuronal number in the basal forebrain after AIE, and expression of cholinergic cells can be recovered with exercise or pharmacological interventions (Vetreno and Crews, 2018; Vetreno et al., 2020; Crews et al., 2021). While neurogenesis could restore cell loss due to cell death, this process is highly confounded by development, and shortly after P10 in rodents, is solely restricted to the subventricular zone and the dentate gyrus of the hippocampus. This suggests that AIE-induced loss and restoration of cholinergic neurons in the basal forebrain is not due to either cell death or spontaneous adult neurogenesis in this region. Rather, the ability to restore ethanol-induced loss of expression of basal forebrain cholinergic neurons in adulthood after adolescent but not perinatal ethanol exposure suggests different mechanisms underlying these long-term changes in cholinergic cell expression. The groundbreaking discovery that cholinergic neurons can be recovered after adolescent but not perinatal ethanol exposure reveals a unique mechanism of neuroplasticity that is centered on epigenetics (Vetreno et al., 2020).

#### Epigenetic Mechanisms Underlying Basal Forebrain Loss of Cholinergic Neurons in Adulthood Following AIE

Significant epigenetic remodeling occurs across adolescence in response to hormonal changes that occur with sexual maturation as well as with environmental maturational events, reflecting a critical window of cellular plasticity related to neuronal circuit maturation during this developmental period (Spear L. P., 2000; Lister et al., 2013; Mychasiuk and Metz, 2016). These modifications are sensitive to ethanol-induced increases in neuroimmune gene expression, and epigenetic modifications induced by ethanol are evident across a variety of brain regions including the amygdala, hippocampus, prefrontal cortex, and basal forebrain (Coleman et al., 2011; Montesinos et al., 2016). Expression of the cholinergic neuronal phenotype requires epigenetic modifications that allow continued expression of several genes necessary for the execution of the synthesis, release, and reuptake of acetylcholine as well as the maintenance of cholinergic projections at target regions. Four of these critical proteins are choline acetyltransferase (ChAT), nerve growth factor (NGF), the high-affinity NGF receptor tropomyosin receptor kinase A (TrkA), and the low-affinity NGF receptor p75NTR, which also can function as a death receptor. Recent findings indicate that adolescent binge ethanol exposure suppresses the cholinergic neuronal phenotype *via* modulation of epigenetic machinery regulating cholinergic genes in both the hippocampus and basal forebrain (as reviewed by (Crews et al., 2019)). In particular, AIE increases histone 3 lysine 9 dimethylation (H3K9me2) by 2.5-fold at the CpG island of the ChAT gene promoter, restricting ChAT gene access to transcriptional machinery, thereby silencing ChAT protein expression as evidenced *via* immunohistochemistry in the basal forebrain (Vetreno et al., 2020). Similarly, AIE increases H3K9me2 occupation 1.7-fold at the CpG island of the TrkA promoter, suggesting reduced gene transcription of this receptor which is critical to NGF-mediated signaling of trophic support. Furthermore, increased H3K9me2 occupation at both of these cholinergic gene promoter regions is associated with increased phosphorylation of NFκB p65 and HMGB1 in basal forebrain cholinergic neurons, suggesting AIE induction of proinflammatory transcription factors may mediate epigenetic suppression of the cholinergic phenotype (Vetreno et al., 2020).

Both HMGB1 and phosphorylation of NFκB p65/p50 have been implicated in pathways that modify epigenetic machinery, although their roles are complex, depending in part on co-activation of other factors. Thus, HMGB1 and NFκB p65/p50 can have distinct functions under normal physiological versus pathological conditions. For example, under normal physiological developmental conditions, nuclear HMGB1 promotes the formation of transcription complexes by overcoming limitations with tightly bent DNA (Crothers, 1993). In contrast, NFκB in combination with HMGB1 can facilitate the formation of repressome complexes wherein HMGB1 binds to G9a, resulting in histone deacetylation and increases in H3K9 and H3K27 methylation that close chromatin suppressing gene transcription (Abhimanyu et al., 2021). This pathway has been best elucidated in studies on peripheral leukocytes, where this pathway functions as a suppressor of proinflammatory response after LPS, reflecting endotoxin tolerance (Gazzar et al., 2009). However, in the brain, regulation of NFκB and HMGB1 complexes are beginning to be elucidated, with evidence mounting that this pathway is particularly important to the suppression of the cholinergic phenotype after AIE (Crews et al., 2021). Of note, prior AIE studies have solely focused on upregulation of NFκB p65; future studies should also investigate p50 due to their somewhat divergent impact on HMGB1-mediated epigenetic modulation of gene transcription.

**FIGURE 4:**
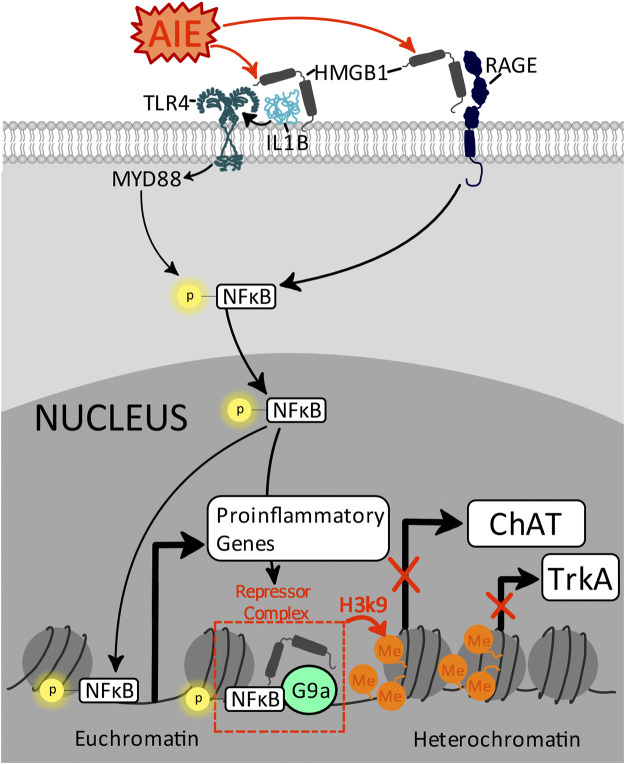
Proposed mechanism by which AIE increases proinflammatory gene transcription and suppresses cholinergic gene transcription through distinct epigenetic modifications. AIE increases extracellular HMGB1 and proinflammatory cytokines IL-1β, which activate TLR4 and RAGE receptors, activating intracellular signaling cascades resulting in the phosphorylation and nuclear translocation of NFĸB p65/50. Within the nucleus, NFĸB is a master regulator of gene transcription with specific effects depending on the types of complexes formed by NFĸB p65/50. For example, NFĸB p65 increases gene transcription of a variety of proinflammatory genes, including TLR4, IL-1β, TNFα, CCL2, and COX-2. However, in the presence of HMGB1, NFĸB p50 forms a repressome complex with G9a (Abhimanyu et al., 2021), driving H3K9 methylation at the ChAT and TrkA promotors, reducing cholinergic gene transcription and suppressing the cholinergic neuronal phenotype (Vetreno et al., 2020; Crews et al., 2021).

Ethanol-induced epigenetic silencing shines light on potential mechanisms for recovery with interventions that reverse this epigenetic silencing (Vetreno et al., 2020), and in fact, both voluntary exercise and galantamine administration reverse epigenetic silencing of basal forebrain cholinergic neurons (Vetreno et al., 2020; Crews et al., 2021). These findings highlight a novel mechanism underlying the perseverance of effects of adolescent binge ethanol on the brain (*see* Figure 4).

#### Adolescent Binge Ethanol Disrupts Cholinergic Signaling in the Neurogenic Niche

Despite the loss of basal forebrain cholinergic neuron phenotype, behaviorally-evoked acetylcholine in the hippocampus during reversal learning in adulthood is slightly, but non-significantly, blunted by prior adolescent binge ethanol exposure (Fernandez and Savage, 2017). This finding needs to be explored further as effects could be distinct across various conditions which evoke acetylcholine release (e.g., cognitive task versus stress versus innate immune challenge; simple task versus complex task), and it is unknown whether these findings extend to females. Regardless, hippocampal cholinergic receptors are adversely impacted by adolescent ethanol exposure (Crews et al., 2016), indicating that even in the absence of overt AIE-induced changes in evoked acetylcholine release in the hippocampus, there may be critical underlying changes in cholinergic signaling dynamics which may be amplified by alterations in cholinergic receptor expression.

Adolescent brain maturation shows developmental reductions in nicotinic cholinergic receptor expression (Coleman et al., 2011). Interestingly, one study found that adolescent binge ethanol exposure accelerates the maturational decline in gene expression for both muscarinic and nicotinic cholinergic receptor subtypes, resulting in even greater reductions in both cholinergic neurons and receptors in adulthood (Coleman et al., 2011). As activation of nicotinic α7 receptors has been identified as an interface in cholinergic anti-inflammatory feedback on microglia (Pavlov et al., 2009; Gnatek et al., 2012; Terrando et al., 2014; Li L. et al., 2019, 7), AIE-induced loss of hippocampal nicotinic α7 receptors could adversely impact microglial ability to regulate innate immune gene expression within the neurogenic niche in adulthood, disrupting the hippocampal microenvironment and facilitating activation of apoptotic cascades. This suggests that AIE-induced loss of cholinergic anti-inflammatory feedback *via* decreased nAChRα7 activation on immunocompetent glia is at the juncture of both persistent inductions in neuroinflammatory signaling cascades, induction of apoptotic executioner caspases, and reductions in survival of adult newborn neurons.

### Reversibility and Treatment Challenges in Models of Adolescent Ethanol Exposure

**FIGURE 5:**
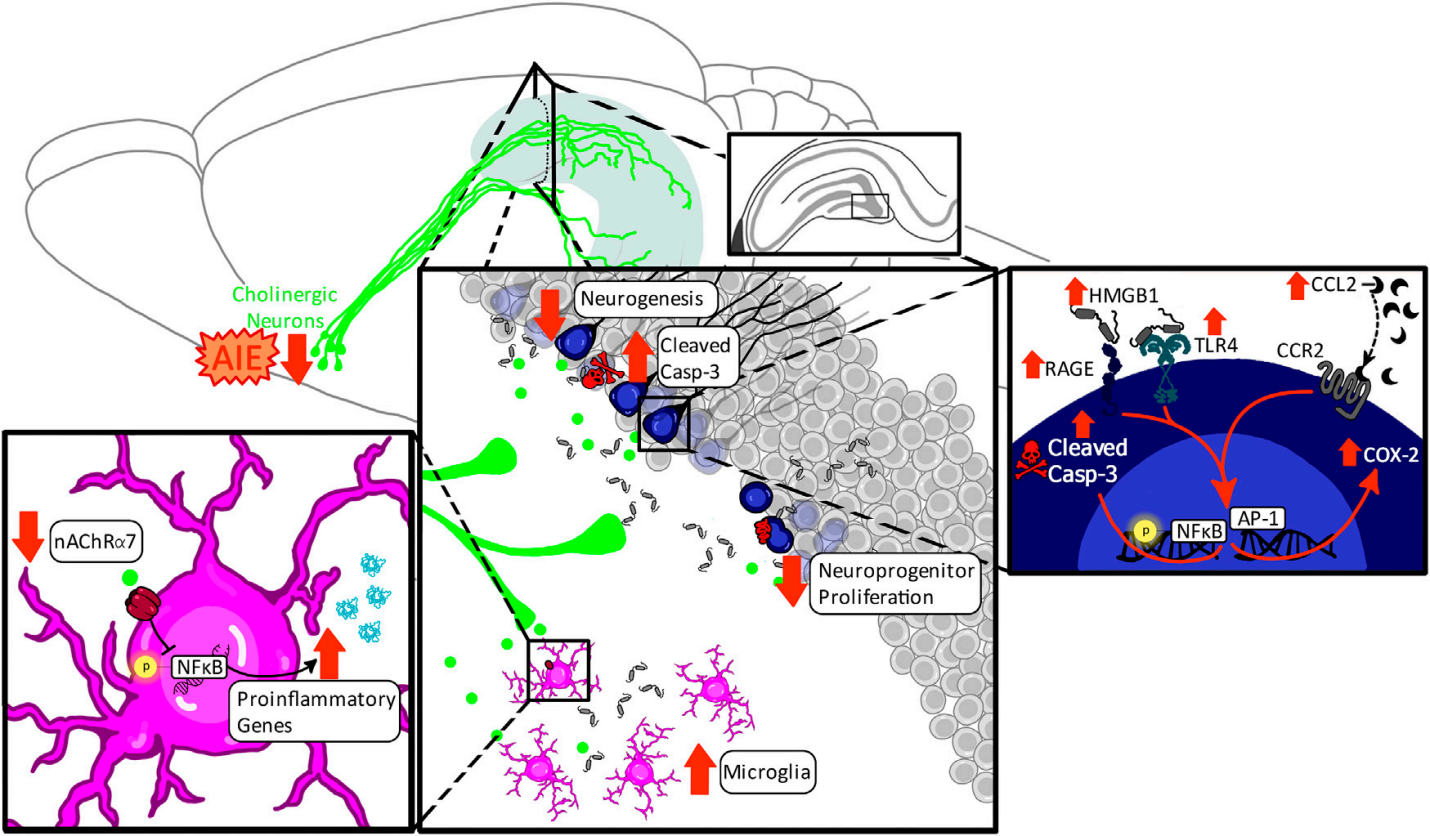
Proposed mechanism underlying the persistent loss of adult hippocampal neurogenesis after AIE. AIE persistently decreases phenotypic expression of cholinergic neurons in the basal forebrain and decreases nAChRα7 expression in the hippocampus, consistent with reduced forebrain-hippocampal cholinergic inhibitory feedback of inflammatory responses. This results in increases in microglia number and proinflammatory microglial phenotypes, and increases hippocampal proinflammatory gene expression in the environmental milieu, including IL-1β, TNFα, and CCL2. Shifts in the hippocampal environmental milieu towards a proinflammatory state has several negative consequences on adult neurogenesis. Increases in extracellular HMGB1 in combination with IL-1β and CCL2 increase neuronal proinflammatory signaling through TLR4/RAGE and CCR2 receptors, respectively. Activation of these receptors results in increases in phosphorylation of NFĸB p65 and translocation to the nucleus where it further potentiates proinflammatory gene expression and induces activation of caspase-3 to initiate cell-death cascades. Increases of caspase-3 in newborn neurons suggest these neurons undergo apoptosis during maturation, resulting in decreased adult hippocampal neurogenesis.

AIE persistent induction of neuroimmune genes as well as reduction in hippocampal neurogenesis corresponds with impairments in a variety of learning and memory tasks, including discrimination learning (Pascual et al., 2007) and reversal learning in males (Crews et al., 2016; Vetreno et al., 2018; Crews et al., 2019). In fact, not only do these neurogenic deficits persist into middle age despite abstinence (Vetreno et al., 2014; Crews et al., 2016), but AIE similarly impairs performance in cognitive flexibility-related tasks in male and female rats long into middle age (Macht et al., 2020b) suggesting that neither AIE-induced deficits in neurogenesis nor AIE-related cognitive flexibility deficits recover with abstinence alone. However, several intervention strategies have emerged that can prevent and/or restore both adult neurogenesis and behavioral flexibility deficits in adulthood in rats exposed to AIE. For example, pre-treatment with the non-steroidal anti-inflammatory drug indomethacin blocks AIE induction of hippocampal inflammatory cascades, restores expression of basal forebrain cholinergic neurons, restores hippocampal neurogenesis (as evidenced by increases in DCX+ immunoreactivity), and restores behavioral deficits (Vetreno and Crews, 2018; Vetreno et al., 2018). The cholinesterase inhibitor galantamine similarly blocks and reverses AIE-induced neuroimmune gene induction as well as blocks and reverses adolescent ethanol-induced loss of neurogenesis (Macht et al., 2021). These findings suggest that AIE-induced reductions in the cholinergic system are a central mechanistic player in both hippocampal neuroinflammatory increases as well as the loss of adult hippocampal neurogenesis and persistence of behavioral flexibility deficits (*see* Figure 5). Adolescent development therefore presents a unique period of vulnerability to the detrimental effects of binge ethanol on adult behavioral and cellular plasticity deficits in the neurogenic niche.

During the adolescent developmental period, changes are governed by alterations in epigenetic regulation of cholinergic and neuroimmune genes, driving an environmental imbalance in the hippocampal environmental milieu which is unfavorable to the successful adult birth, maturation, and integration of newborn hippocampal neurons. This suggests that in the absence of intervention, both adolescent ethanol-induced loss of neurogenesis (Vetreno and Crews, 2015), basal forebrain cholinergic neuron expression, and cognitive-behavioral deficits (Macht et al., 2020b) persist long into adulthood, suggesting a possible permanence of these neurodevelopmental alterations. This highlights the necessity for active interventions following adolescent ethanol exposure to restore both hippocampal neurogenesis and cognitive function. Surprisingly, as the loss of basal forebrain cholinergic neurons is mediated by epigenetic mechanisms, restoration of cholinergic function after adolescent ethanol has the potential to be restored by reversing epigenetic suppression of the cholinergic phenotype. Collectively, these results suggest enhanced sensitivity of neuroprogenitors to ethanol during adolescence. The persistence of these effects suggest that this period confers a unique developmental susceptibility to ethanol with potential long-lasting consequences on hippocampal neurocircuitry and behavior. However, the reversibility of these factors through voluntary exercise gives hope for interventions aimed at restoring these deficits in adulthood.

### Adult Alcohol Use Disorder and Consequences Revealed by Preclinical Models

Alcohol use disorder (AUD) is highly prevalent in Western society, with estimates of up to 29% of adults suffering from AUD at some point in their lifetime (Grant et al., 2015). This disorder is characterized by patterns of high levels of alcohol intake despite adverse social, occupational, economic, and health consequences. While AUD has historically been more prevalent in adult males, prevalence of AUD in adult females has been rapidly rising over the last decades (Roberta et al., 2017). The issue of AUD has become increasingly prevalent in the face of the COVID-19 pandemic as studies estimate that ∼24% of individuals reported increases in alcohol intake irrespective of prior diagnosis of AUD, and 17% of previously abstinent individuals with AUD relapsed during pandemic lockdowns (Kim et al., 2020). These increases in adult binge drinking escalate over time, with 1.19-fold greater odds of individuals binge drinking for every week at home (Weerakoon et al., 2021). Adult binge alcohol consumption is concerning as it results in a host of neurological changes and damage, including increases in neuroinflammatory factors as well as decreases in hippocampal neurogenesis and some subtle changes in the central cholinergic system. Therefore, understanding the long-term neurobiological consequences to binge drinking in adulthood is critical to intervention strategies, and of escalating importance in today’s society.

### Adult Ethanol Exposure Transiently Impairs Survival of Hippocampal Neuroprogenitors and Neurogenesis

Although neurogenesis was originally thought to be isolated to the neonate, Altman and Das (Altman and Das, 1965) revolutionized this view with their 1965 seminal findings. It is now estimated that up to 6% of granular cell neurons are replaced every month in adulthood in rodents as a result of hippocampal neurogenesis (Cameron and Mckay, 2001). Although the prevalence and function of adult neurogenesis in humans is an ongoing scientific debate centered on technical discrepancies (for review *see* (Kempermann et al., 2018)), rodents have routinely demonstrated quantifiable neurogenesis in adulthood with emerging work centered instead on elucidating the functional roles of neurogenesis in relation to hippocampal circuitry and behavior (van Praag et al., 2002; Clelland et al., 2009; Garthe and Kempermann, 2013). Collectively, results seem to indicate that these new neurons play critical roles in hippocampal physiology and neuronal plasticity, and as with other developmental periods, adult newborn neurons are sensitive to the effects of ethanol.

Preclinical studies indicate that adult chronic binge ethanol exposure decreases hippocampal neuroprogenitor cell proliferation and neurogenesis (Nixon and Crews, 2002) even in models of moderate alcohol blood ethanol concentrations (Anderson et al., 2012), although the severity of effects increases with prolonged dependence (Richardson et al., 2009). However, these results become more complex when comparing these models of adult AUD to adolescent intermittent exposure paradigms as there are important distinctions in the models used, particularly in relation to ethanol dependence and withdrawal symptoms. For example, 4-day binge and liquid diet paradigms which model human AUD result in physical dependence to ethanol in conjunction with pronounced withdrawal symptoms (Morris et al., 2010). Models of AIE do not result in dependence or severe withdrawal symptoms. In fact, when ethanol is administered intermittently across adulthood (P70-90), there are no reductions in hippocampal neurogenesis, unlike in adolescent intermittent ethanol models (Broadwater et al., 2014). This suggests that intermittent ethanol exposure in adulthood is not sufficiently severe to induce long-term neurogenic deficits, and instead models of AUD, which result in physical dependence and/or severe withdrawal systems, are necessary for inducing deficits in adult hippocampal neurogenesis after adult ethanol exposure (Table 1).

**FIGURE 6:**
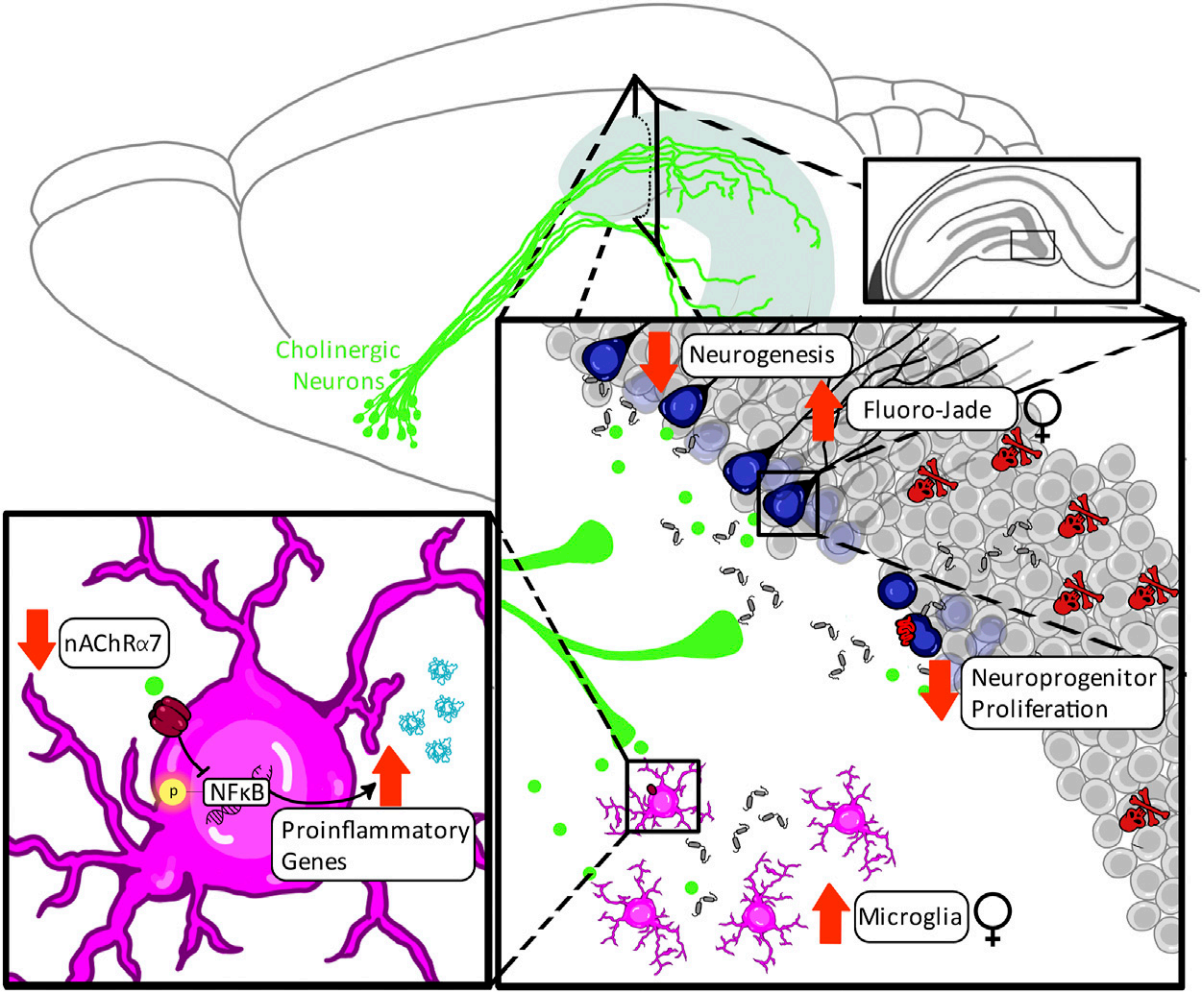
Proposed model of chronic adult ethanol impact on hippocampal neuroinflammation and neurogenesis. The adult neurogenic niche is sensitive to neuroinflammatory insults with females being particularly sensitive to these effects. Heightened sensitivity to inflammatory factors may be mediated by reductions in nicotinic α7 receptor expression in the adult hippocampus (Robles and Sabriá, 2008), resulting in poor cholinergic regulation of neuroinflammation that is independent from overarching loss of cholinergic cells. This ethanol-induced disruption in the hippocampal environmental milieu increases necrotic cell death in the granule cell layer of the hippocampus with females being particularly sensitive to ethanol-induced granule cell loss in adulthood (West et al., 2019). However, the impact of chronic ethanol in adulthood on the neurogenic niche is complex. Non-dependent or dependent, but short-term binge ethanol models in adulthood result in either no changes in adult neurogenesis or a transient burst-like increase in neurogenesis that is associated acutely with the withdrawal period (Nixon and Crews, 2004; West et al., 2019). In contrast, long-term dependence models reveal reductions in hippocampal neurogenesis, which are primarily driven by ethanol-induced reductions in the neuroprogenitor pool (Richardson et al., 2009; Stevenson et al., 2009).

Adult ethanol-induced suppression of hippocampal cell proliferation is further complicated by withdrawal periods, as there is some evidence for a brief burst in cell proliferation 1 week into abstinence (Nixon and Crews, 2004). However, this effect appears to be insufficient to recover neurogenesis as 2 weeks of abstinence is insufficient to recover ethanol-induced loss of DCX immunoreactivity after 1 month of ethanol self-administration (Stevenson et al., 2009). Moreover, there is some evidence that loss of neuroprogenitor proliferation after binge ethanol is more dramatic in females than males (Maynard et al., 2018), and that this decrease in female progenitors is similarly due to greater sensitivity to ethanol’s impairment in the availability of trophic support. Unlike perinatal or adolescent binge alcohol exposure, adult binge alcohol exposure does not increase cellular apoptosis in the adult dentate gyrus (Obernier et al., 2002), suggesting a divergence of mechanisms between developmental and adult alcohol impairments in hippocampal neurogenesis. However, adult binge alcohol does induce necrotic cell death in the hippocampus with females again being particularly sensitive to this effect, resulting in greater loss in hippocampal cell number after binge alcohol specifically in females (Maynard et al., 2018). This suggests that although adult binge ethanol exposure reduces neurogenesis in males and females, this effect could be particularly devastating in females due to an inability to repopulate the granular cell layer over time. In support of this, some recent findings suggest that recovery of neurogenesis after recurrent binge adult alcohol exposure by short-term voluntary wheel running (3 days) is insufficient to recover granular layer cell loss in females (West et al., 2019). Therefore, alcohol-induced granular cell layer loss in females may require more prolonged or intensive therapeutic intervention strategies (for summary, *see* Figure 6).

### Modest Changes in Cholinergic Innervation of the Hippocampus After Ethanol Exposure May Underlie Hypersensitivity to Inflammatory Challenges in Adulthood

The basal forebrain cholinergic system is profoundly affected by ethanol during early development, with both perinatal and AIE exposure reducing basal forebrain cholinergic neuron expression in addition to decreasing cholinergic regulation of the hippocampus. However, adult alcohol exposure does not produce as rapid or robust an effect on the basal forebrain cholinergic system. Just as intermittent ethanol exposure across adolescence but not adulthood produces deficits in adult hippocampal neurogenesis (Vetreno et al., 2018), adolescent but not adult intermittent ethanol exposure reduces basal forebrain ChAT immunoreactivity (Vetreno et al., 2014). Even more dramatically, 20 days of repeated adult binge ethanol exposure in adulthood is still insufficient to reduce basal forebrain cholinergic neuron numbers (Vetreno et al., 2014). Only studies that have employed a forced sole-source ethanol drinking paradigm of 12+ weeks have shown ethanol-reduced basal forebrain cholinergic neuron expression in adults (Arendt et al., 1988b), indicating that these cells are far less susceptible to ethanol-induced damage in adulthood. However, chronic adult ethanol consumption does impact axonal projections, with evidence of decreased cholinergic innervation of the hippocampus (Cadete-Leite et al., 1995). This suggests that even in the absence of reductions in cholinergic cell numbers, chronic adult ethanol exposure can reduce cholinergic projections resulting in impaired function at target regions, including the hippocampus. In support of this, in *in vivo* microdialysis studies in adults after long-term 3-month ethanol exposure in drinking water, acetylcholine efflux in the hippocampus was significantly reduced by 57%, relative to controls (Casamenti et al., 1993). This effect was reversible with abstinence, suggesting a greater degree of plasticity in cholinergic systems in adulthood, and increased recoverability following ethanol exposure.

Chronic ethanol exposure in adulthood can also disrupt cholinergic receptors, but this finding is complex and largely depends on subtype. For example, 10 weeks of ethanol consumption decreases hippocampal nicotinic receptor expression independently from altering binding affinity (Robles and Sabriá, 2008). In contrast, rodent models using a 28-week liquid diet of ethanol have not reported alterations in number of muscarinic receptor subtypes in the hippocampus (Rothberg et al., 1993, Rothberg et al., 1996). Despite evidence suggesting that there is no impact of ethanol on binding affinity or number of muscarinic receptor subtype, models of chronic ethanol exposure in adulthood do impair acetylcholine-mediated evoked electrophysiological post-synaptic potential responses in pyramidal cells of the CA1 region of the hippocampus, suggesting impaired cholinergic function in this region (Rothberg and Hunter, 1991). Cholinergic activation of pyramidal cells is thought to be mediated through muscarinic receptors, as these effects can be blocked by the muscarinic atropine but not nicotinic agonists (Cole and Nicoll, 1984; Valentino and Dingledine, 2021). This reduced hippocampal cellular responsivity to acetylcholine is postulated to be due to impaired intracellular transduction mechanisms following cholinergic muscarinic receptor activation, suggesting that chronic ethanol may alter muscarinic receptor function in the absence of overarching loss of receptor number. As proliferation of neuroprogenitor pools is dependent on cholinergic activation of muscarinic receptors and subsequent mobilization of intracellular signaling cascades (Ma et al., 2000), these findings suggest that alterations in responsivity to acetylcholine in the hippocampus could underlie some of the deficits in neuroprogenitor proliferation evidenced after chronic ethanol exposure in adulthood.

The loss of hippocampal nicotinic receptor expression could underlie sensitivity to neuroinflammatory induction after chronic ethanol in adulthood, as microglial nicotinic α7 receptors play critical negative feedback role for proinflammatory cascades. Thus, loss of activation at nicotinic α7 receptors is suggestive of a loss of anti-inflammatory feedback and chronic induction of proinflammatory signaling cascades in the brain of individuals with AUD. In support of this concept, an increase in neuroinflammation in post-mortem human brains with AUD is one of the most prevalent clinical findings (Coleman and Crews, 2018), and preclinical models further suggest that neuroinflammation is tightly coupled to continuation of drinking behaviors in nonhuman primates (Beattie et al., 2018). Preclinical models have replicated these results: 6 months of ethanol treatment followed by 2 months of withdrawal in male rats resulted in long-term induction on the activated microglial marker CD11b and the cytokine IL-15 (Cruz et al., 2017). More recent evidence suggests that females may be even more sensitive to this effect as a 4-day binge is sufficient to increase microglial number and frequency of activated phenotypes, indicated by increases in microglia expressing major histocompatibility complex II (MHC II) in the hippocampus of female but not male rats (Barton et al., 2017). These findings support a growing body of clinical and preclinical findings indicating that females may be more sensitive to ethanol-induced damage in adulthood (Alfonso-Loeches et al., 2013).

The effect of ethanol on hippocampal neuroinflammation can be magnified under conditions which challenge the immune system, such as a LPS or polyI:C innate immune challenge. Ten days of intragastric administration of ethanol in mice potentiates proinflammatory responses in brain to a lipopolysaccharide-TLR4 innate immune challenge which included CCL2, COX-2, gp91^phox^ NADPH oxidase subunit, TNFα, and IL-1β as well as greater morphological changes in microglia (Qin et al., 2008). Similarly, a polyI:C-TLR3 challenge after 10 days of ethanol administration also results in an exaggerated increase in the microglial marker ionized calcium binding adaptor molecule 1 (Iba1) in the dentate gyrus of the hippocampus (Qin and Crews, 2012). These neuroinflammatory inductions after ethanol were also associated with reductions in the newborn neuronal marker DCX and greater induction of necrotic and apoptotic cell death in the hippocampus, further highlighting that induction of neuroinflammation, sensitivity to activation of cell death cascades, and loss of neurogenesis are tightly coupled. In fact, in studies of rat hippocampal brain slice cultures, blocking IL-1β with either an antagonist or neutralizing antibody blocked adult ethanol inhibition of hippocampal neurogenesis (Zou and Crews, 2012). Neuroimmune signaling involves feed-forward processes that increase expression of proinflammatory agonists and receptors, particularly TLR receptors. Inhibition of proinflammatory signaling at multiple components of the amplification process are effective. These findings highlight that neuroinflammation is a particularly crucial mediator of neurogenic deficits in adulthood.

### Reversibility and Treatment Challenges in Models of Adult Alcohol Use Disorder

Therapeutics under investigation for AUD often exhibit anti-inflammatory components. Given the critical role of the cholinergic system in regulating anti-inflammatory feedback, it is unsurprising that many of these restorative interventions center around the cholinergic systems. Rodent and human studies suggest successful therapeutic targets at both cholinergic muscarinic M4 receptors (Walker et al., 2020) and nicotinic α7 and α7β3 receptors through varenicline (Witkiewitz et al., 2019), and since the late 1980s, rat models of AUD have even been treated with cholinergic-rich brain transplants (Arendt et al., 1988a). While the majority of therapies for AUD focus on reducing relapse behaviors and overall ethanol intake, the ability to reverse molecular cascades involved in cognitive deficits after long-term ethanol use is another important consideration. The mechanisms underlying the efficacy of cholinergic-based therapeutics continue to be investigated for use in alcohol-related disorders, but an emerging theme is their regulation of ethanol-induced neuroinflammation. Hippocampal neuroinflammation is a critical long-term consequence of adult ethanol intake, partially through its mediation of caspase-activated apoptotic cascades and reductive consequences on hippocampal neurogenesis (Liu et al., 2021). In fact, targeting cholinergic systems in adulthood after ethanol use may be effective not through direct increases in activation of cholinergic circuits, but rather indirectly through their mediating factors on both neuroinflammation and neurogenesis. To emphasize this point, targeting ethanol’s induction of IL-1β is sufficient for restoration of hippocampal neurogenesis in adulthood in rodents (Zou and Crews, 2012). This suggests that ethanol induction of neuroinflammatory markers and hypersensitivity of inflammatory systems in adulthood is a central mediator to the loss of hippocampal neurogenesis.

These mechanisms linking neuroinflammation and neurogenesis are particularly important when examining sex differences as human females are more likely than men to experience organ damage following long-term alcohol use, including greater brain damage in general and more specifically loss of hippocampal volume (Agartz et al., 1999; Hommer et al., 2001; Mann et al., 2005; Sharrett-Field et al., 2013), and are more likely to need active interventions beyond abstinence than males. For example, preclinical models indicate that abstinence is insufficient for granule cell layer recovery in females in the absence of an intervention such as voluntary exercise (Maynard and Leasure, 2013). Voluntary exercise after adult ethanol exposure restores the proinflammatory-trophic balance in the hippocampal environmental milieu – a balance which exhibits greater ethanol-induced disruptions in females than males (Maynard et al., 2018). These alarming emerging findings suggest that examining the mechanisms for reversing ethanol-related brain damage in females is becoming an increasingly critical area of investigation as females close the gender gap in AUDs.

## Conclusions: Development of Interacting Physiological Systems Mediate the Persistent Neuropathology Induced by Ethanol Across the Lifespan

The molecular mechanisms of ethanol-related neuronal damage and related intervention strategies circulate around the cholinergic system, neuroinflammation, and the mechanistic mediators of these systems on hippocampal neurogenesis regardless of developmental window, suggestive of certain central commonalities within the effects of ethanol on these systems. However, across the neurodevelopmental landscape, while the mechanisms may overarchingly be consistent, various systems display differing sensitivities in the magnitude and reversibility of ethanol-related disruptions. For example, in models of FASD, ethanol produces seemingly irreversible deficits in the cholinergic system, and one of the key factors in treatment for FASD focuses on preventing rather than reversing this neuronal damage. If unmitigated, the irrecoverable loss of cholinergic neurons is a hindrance to the functional recovery of ethanol-related damage, and later life consequences of this disruption include greater neuroinflammatory responses and decreases in neurogenesis when neural systems are challenged. As females tend to be diagnosed with FASD later than males, this suggests that intervention strategies may be particularly difficult for this population as females have a greater likelihood of missing early intervention therapeutic windows.

In contrast, adolescence is marked by epigenetic-mediated suppression of basal forebrain cholinergic phenotypes, which is exciting from a therapeutic standpoint as reversal of epigenetic machinery in cholinergic neurons similarly restores their phenotypic expression, which consequently also restores the hippocampal neuroinflammatory-trophic balance as well as ethanol-mediated deficits in hippocampal neurogenesis. This balance between persistence and reversibility after adolescent ethanol exposure highlights a shift in the mechanisms driving ethanol-induced loss of hippocampal neurogenesis and neurocognitive impairments from the cholinergic system as the central mediator to neuroimmune dysregulation playing a greater role.

This shift becomes more apparent in adulthood, where hippocampal neuroimmune induction in both preclinical models and in human postmortem tissue from individuals diagnosed with AUD persists long after ethanol exposure and seem to drive long-term cellular consequences, including hippocampal damage and deficits in neurogenesis. While abstinence reverses some of these deficits in males, females exhibit greater sensitivity to ethanol-related brain damage, with emerging studies finding that females require more aggressive intervention strategies than males.

The shifts in vulnerability of cholinergic versus neuroimmune systems over development suggests that efficacy of interventions aimed at restoring ethanol-related damage to hippocampal neurogenesis and cognitive function must take a developmental approach. However, a limitation of preclinical models of FASD, adolescent binge drinking, and AUD is that often alcohol exposure in humans is not isolated to a single developmental period. In fact, prior exposure to ethanol either *in utero* or in adolescence increases drinking behaviors at later developmental windows (Moore and Riley, 2015; Crews et al., 2019). Thus, an individual with AUD is more likely to have engaged in binge drinking during adolescence. Similarly, an individual who drinks in adolescence is likely to continue drinking into adulthood, and an individual with FASD is significantly more likely to misuse alcohol as adolescents and develop AUD as an adult (Baer et al., 2003; Alati et al., 2008). This double- or even triple hit of ethanol-mediated neuronal disruption over time adds layers of complication to the impact of ethanol on neural systems in clinical populations, and remains an important future direction in preclinical research. For example, the persistent decrease in cholinergic systems after perinatal ethanol exposure may create a vulnerability for even more dramatic neuroimmune induction and loss of neurogenesis following subsequent adolescent binge drinking. Sex differences in vulnerability to ethanol’s disruption of developing neural and immune systems may similarly compound over the lifespan, shifting the severity, perseverance, and/or reversibility of ethanol’s molecular and behavioral consequences, suggesting that the focus of intervention strategies may need to shift over time across sexes but also even within individuals depending on their age and history of exposure.
